# Supplementary material for: Rapid formation of non-spatial hippocampal representations consistent with behavioral timescale synaptic plasticity is modulated by entorhinal input
Source: Nat Commun. 2026 Apr 10;17:5098. doi: 10.1038/s41467-026-71503-y (PMC13247236; doi:10.1038/s41467-026-71503-y)
Supplement: Supplementary file 1 — Supplementary Information [file 41467_2026_71503_MOESM1_ESM.pdf]

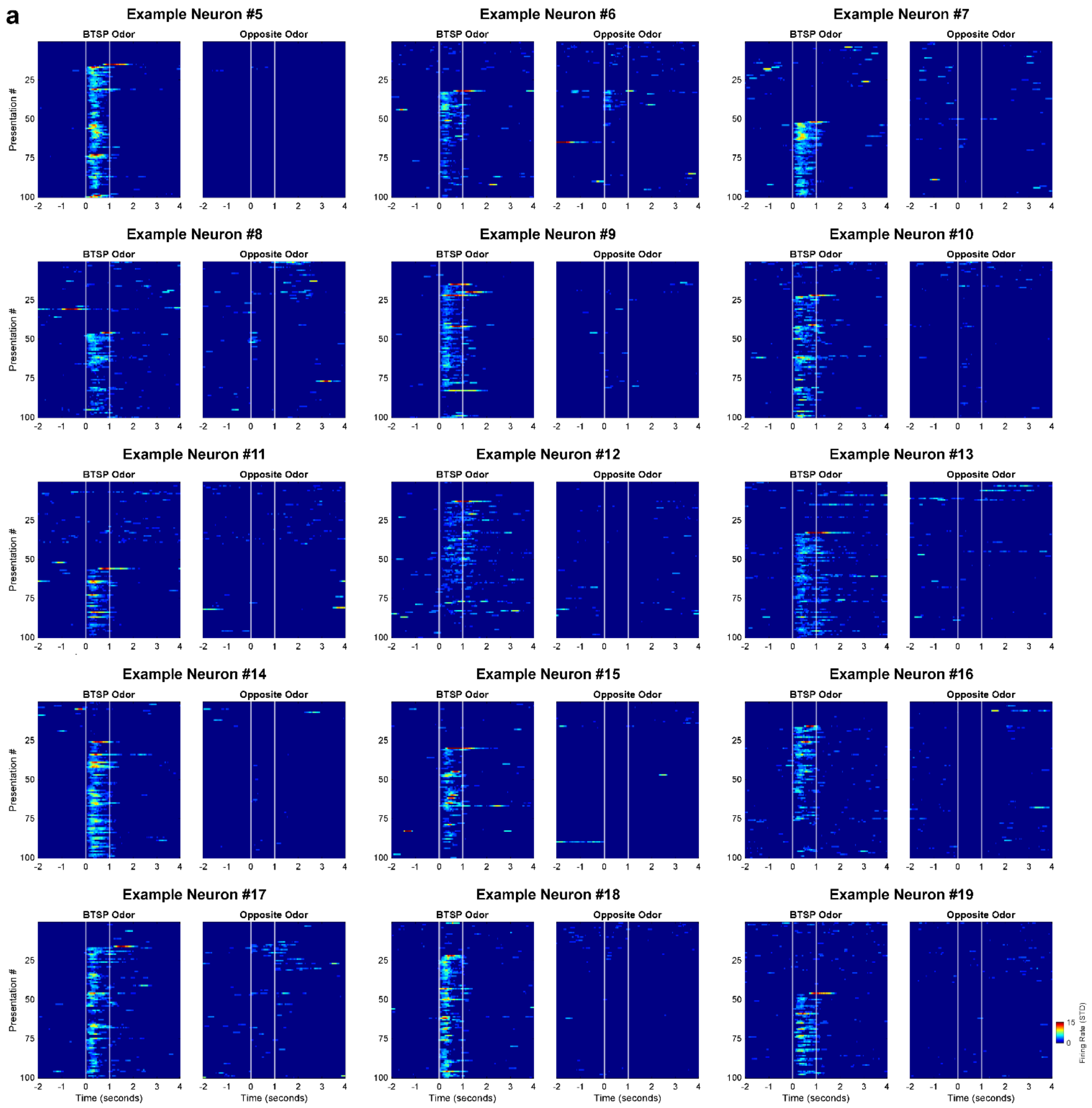

Supplementary Figure S1: Additional examples of 'BTSP-like' events. a, 15 more example neurons showing 'BTSP-like' events.

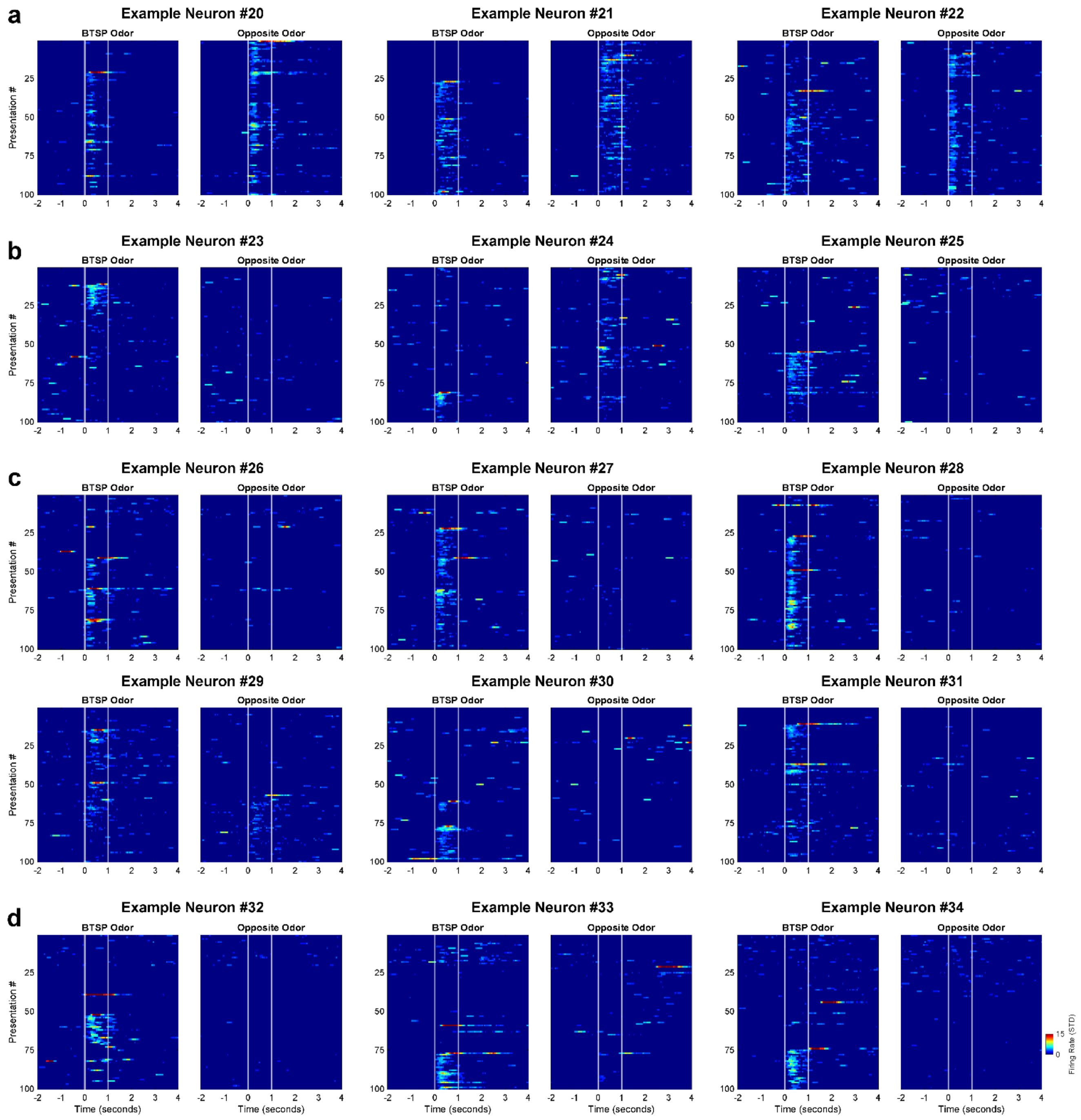

Supplementary Figure S2: More unique examples of 'BTSP-like' events. a, 3 examples of an odor-selective neuron becoming non-selective because of the new BTSP induced field. **b,** 3 examples of neurons that formed an odor-field after a 'plateau-like' event, but the field faded quickly. **c,** 6 examples of neurons with multiple 'plateau-like' events that seem to reinforce the odor-field. **d,** 3 examples of failed 'plateau-like' events followed by successful ones.

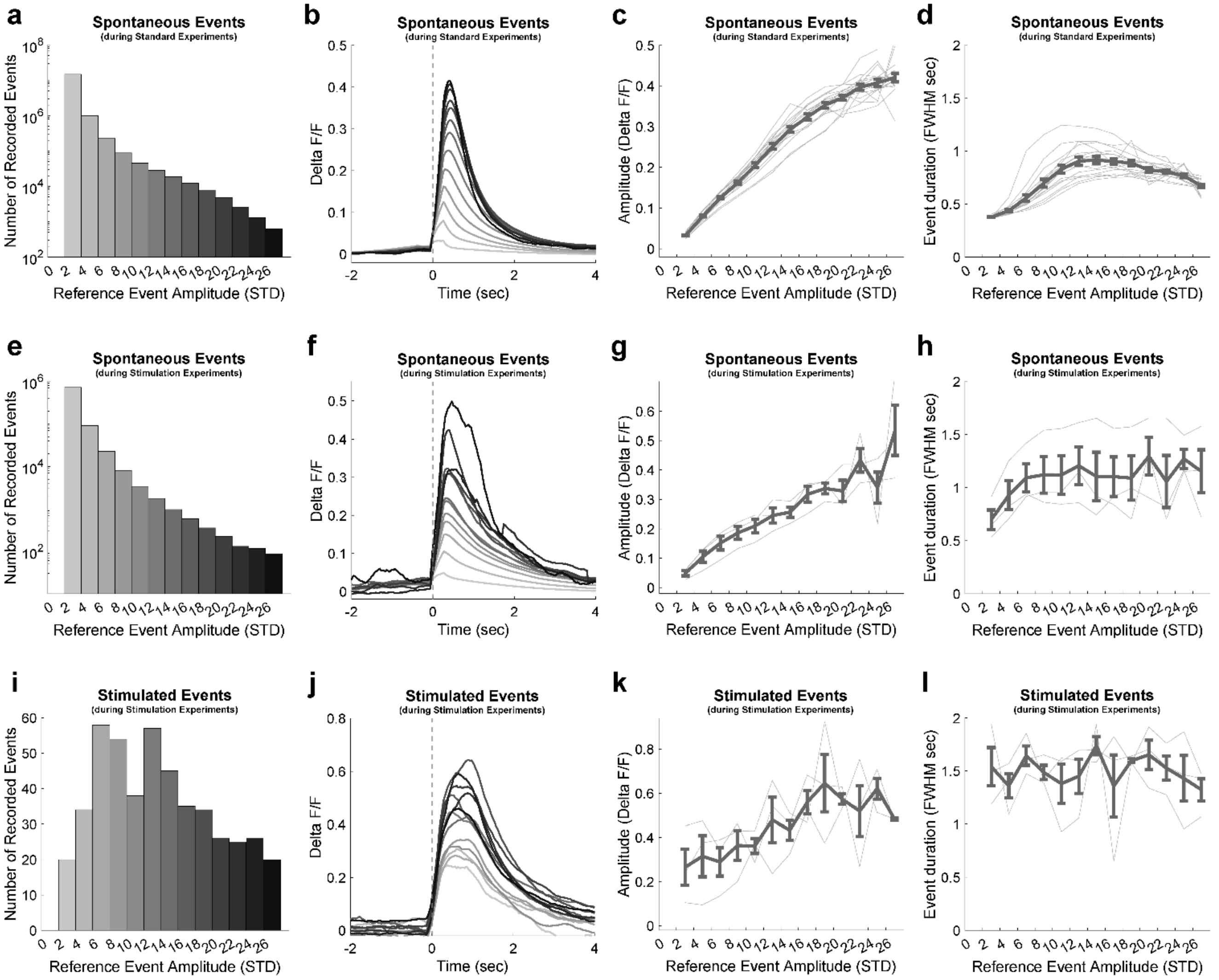

Supplementary Figure S3: Shapes and sizes of 'plateau-like' events. a, sum of total number of recorded deconvolved spontaneous events in the 17 animals from Figure 1 (136 recording sessions). Logarithmic scale shows rare occurrence of large events compared to smaller events. **b,** Delta F/F traces for all events binned into the same deconvolved STD values as in (a) and aligned to the start of the deconvolved event. Colors are matched with (a). **c,** Peak amplitude of average Delta F/F traces from (b). Each line represents one of the 17 animals. Error bar line plot represents mean and SEM. **d,** Full-width at half-maximum (FWHM) for average Delta F/F traces from (b). Lines and error bars are the same as (c). **e-h,** Same as (a-d), but for all spontaneous events recorded during holographic optogenetics experiments from Figure 2 (3 animals, 16 recording sessions). **i-l**, same as (e-h), but only for optogenetically stimulated events from Figure 2. Standard y-axis for (i) instead of logarithmic.

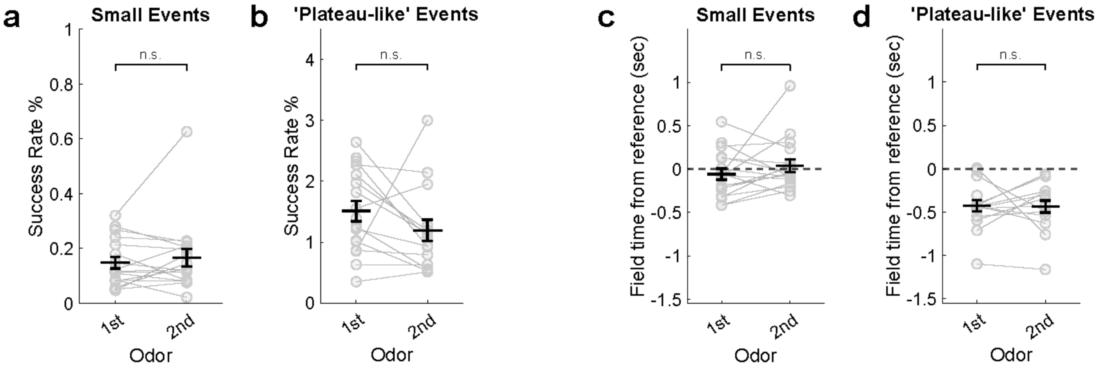

Supplementary Figure S4: 'BTSP-like' events are similar for first and second odor presentation periods. Detection analysis was re-run using either the first odors or second odor presentations. Complementing previous work in the lab using the same task[19], we find no difference between the two odors. **a,** Success rates of small events are not different between first and second odors (two-tailed paired t-test, p = 0.4342). Dots and lines represent each animal (17 animals, which are the average for each of their 8 recording sessions). Error bars represent the mean and SEM. **b,** Same as (a), but for 'plateau-like' events, which are also not different (two-tailed paired t-test, p = 0.2303). **c,** Similar to (a), but calculating the field time from reference as in Figure 1i for all newly formed odor-fields (two-tailed paired t-test, p = 0.2429). **d,** same as (c), but for 'plateau-like' events (paired t-test, p = 0.7371).

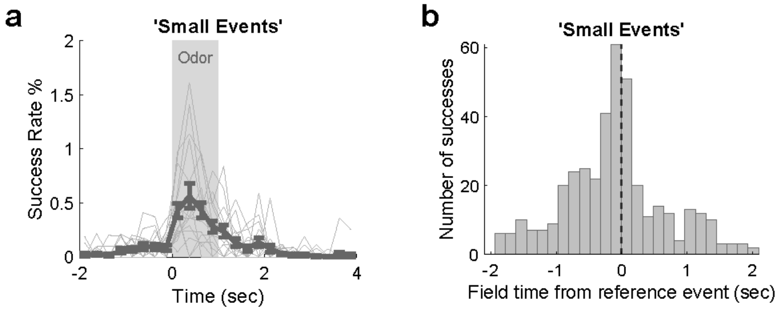

Supplementary Figure S5: Small events were not BTSP. a, Supplementing Fig. 1j showing success rate was very low for small events. These represent randomness of non-BTSP events passing criteria for BTSP. **b,** Supplementing Fig. 1k showing that small events did not have asymmetrical formation that was seen for 'plateau-like' events (n=396).

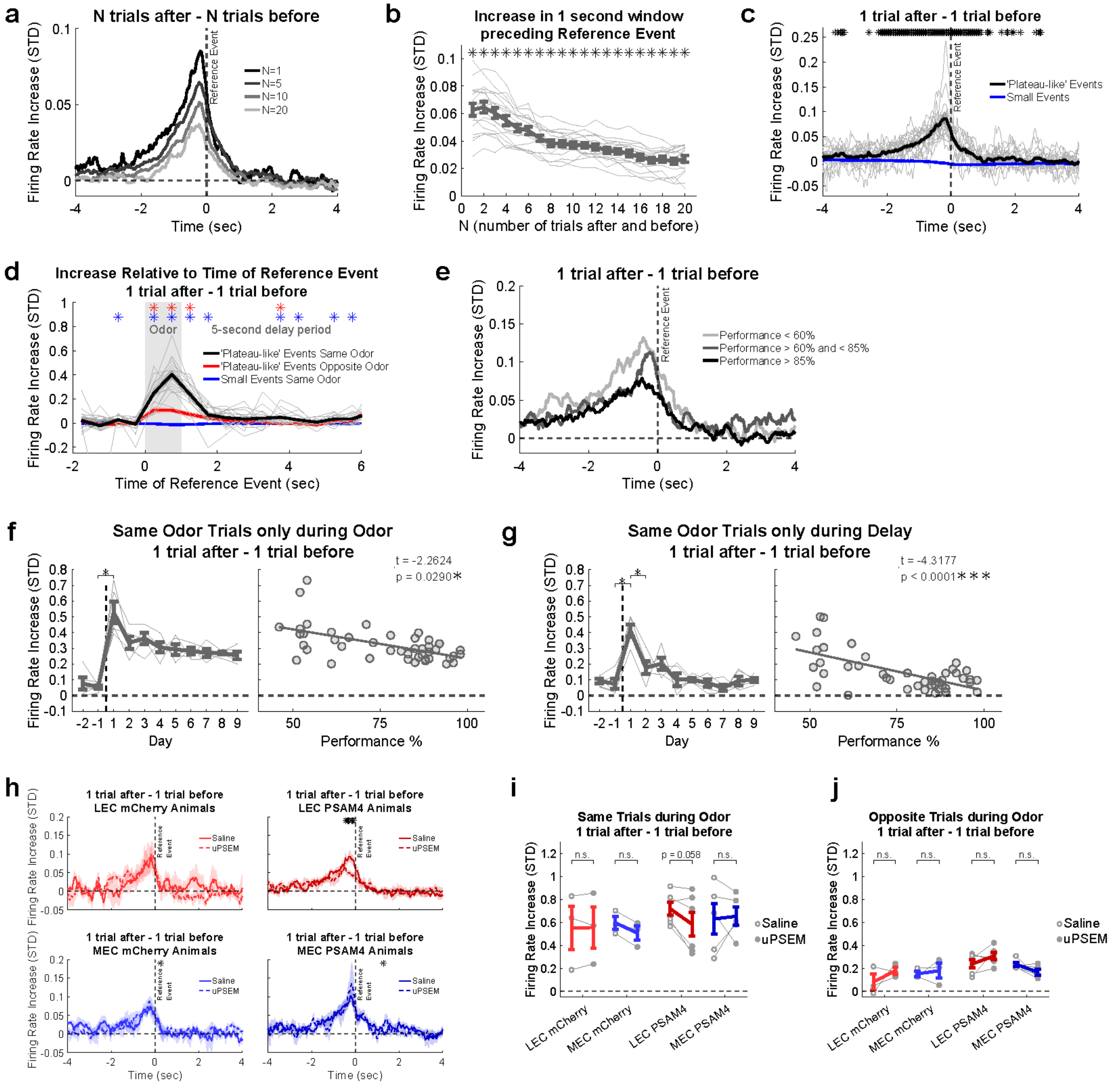

Supplementary Figure S6: Firing rate increase effects were found at a range of number of trials after and before. **a,** Supplementing Fig. 1l to show the firing rate increase ramp with 4 different number of trials (N) after and before. **b,** Calculated as the average of the ramp between -1 and 0 seconds in trial-time, plotted as a function of N. Statistics are one-sample t-tests with each N having a $p < 0.05$ (corrected for multiple comparisons using the Benjamini-Hochberg procedure). N = 10 was chosen for the main figures because it minimized variability. N = 1 has the largest effect but largest variability. **c,** Same as Fig. 1l but for only 1 trial after and 1 trial before. **d,** Same as Fig. 1n. **e,** Same as Fig. 3f. **f,** Same as Fig. 3g. Visualizations and statistics are the same as Fig. 3g. Additional right panel statistics from linear regression model can be found in Supp. Table. 6. **g,** Same as Fig. 3i. Visualizations and statistics are the same as Fig. 3i. Additional right panel statistics from linear regression model can be found in Supp. Table. 7. **h,** Same as Fig. 4d. **i,** Same as Fig. 4e. LEC PSAM mean difference 95% confidence interval = -0.006 to 0.28. **j,** Same as Fig. 4f.

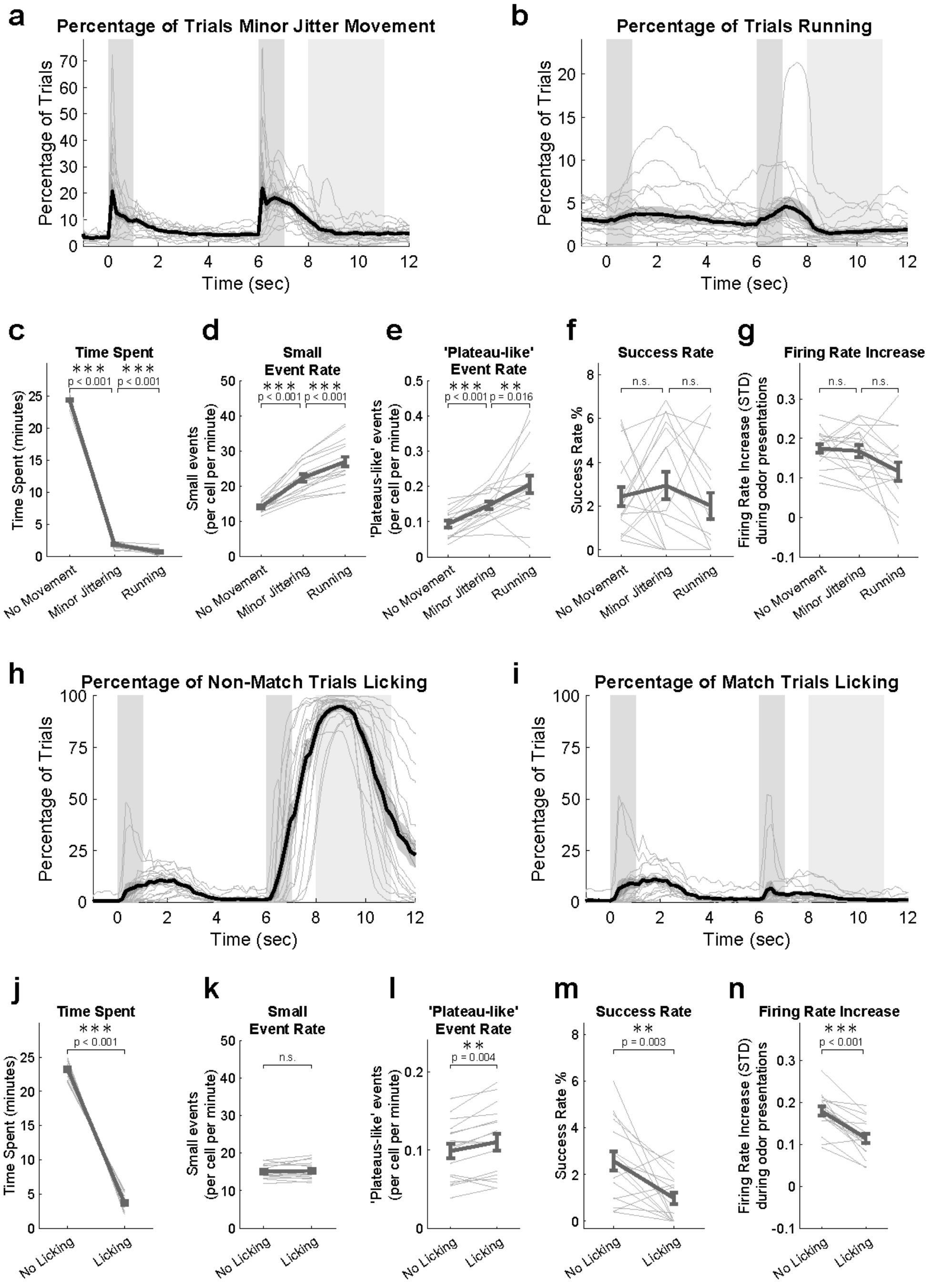

Supplementary Figure S7: All types of movements increased neural activity but had no effect on BTSP. a, Percentage of trials with a minor jitter movement at each bin in the trial. Minor jitter movement is defined as a significant positive or negative treadmill movement that is less than one second in duration. Individual thin gray lines represent each of the 17 animals (which are the average for each of their 8 recording sessions. **b,** Percentage of trials with a bout of running at each bin in the trial. Running is defined as a significant negative treadmill movement (ball rolling backwards with forward mouse movement) that is at least one second in duration. **c,** Percentage of trial-time of the 3 types of movement on treadmill. Total time is 27 minutes (100 trials of 16.2 seconds - 2 baseline minutes and 14.2 seconds after onset of first odor). Individual thin gray lines represent each of the 17 animals (which are the average for each of their 8 recording sessions. Statistics are paired t-tests. **d,** Number of events with amplitudes less than 10 STD per neuron per minute of recording. **e,** Number of events with amplitudes greater than 10 STD per neuron per minute. **f,** Success rate of 'plateau-like' events that occurred

during the 3 types of movements. **g,** Firing rate increase of ‘plateau-like’ events. **h,** Same as (a) for licking in non-match trials. **i,** Same as (h) for match trials. **j-n,** Same as (c-g), but for licking on all trial types.

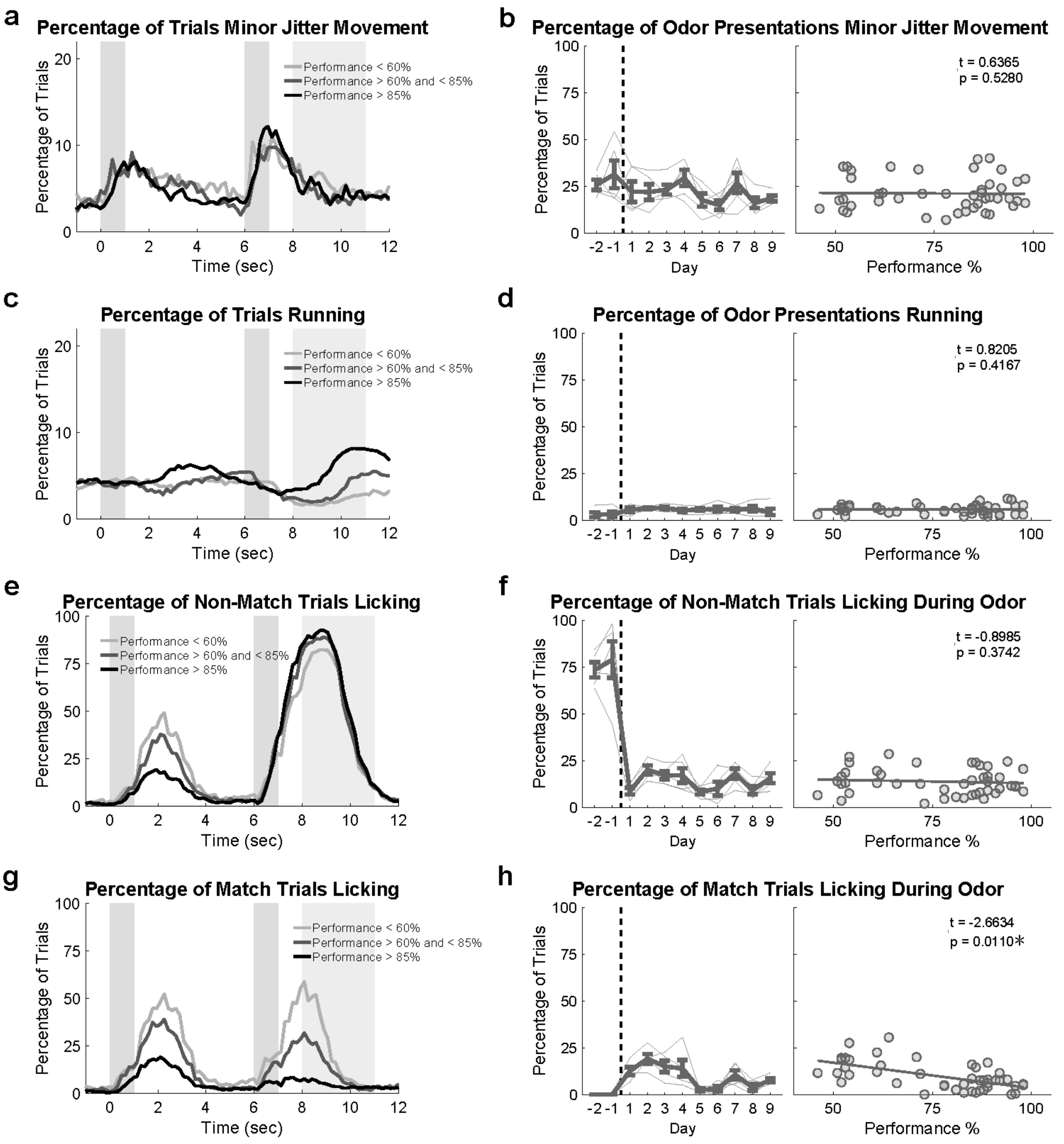

Supplementary Figure S8: Movements do not change across learning during odor presentations. a, Similar to Supp. Fig. S7a, but for learning animals and split by 3 levels of performance. **b,** Percentage of odor presentations with minor jitter movements did not differ across days and performance. Visualizations and statistics are the same as similar panels in Figure 3. Additional right panel statistics from linear regression model can be found in Supp. Table. 8. **c-d,** Same as (a-b), but for running bouts. Visualizations and statistics are the same as similar panels in Figure 3. Additional right panel statistics from linear regression model can be found in Supp. Table. 9. **e-f,** Same as (a-b), but for licking in non-match trials. Visualizations and statistics are the same as similar panels in Figure 3. Additional right panel statistics from linear regression model can be found in Supp. Table. 10. **g-h,** Same as (e-f), but for match trials. Visualizations and statistics are the same as similar panels in Figure 3. Additional right panel statistics from linear regression model can be found in Supp. Table. 11. The effect is related to learning to refrain from licking on these match trials, but it cannot explain any effects related to BTSP.

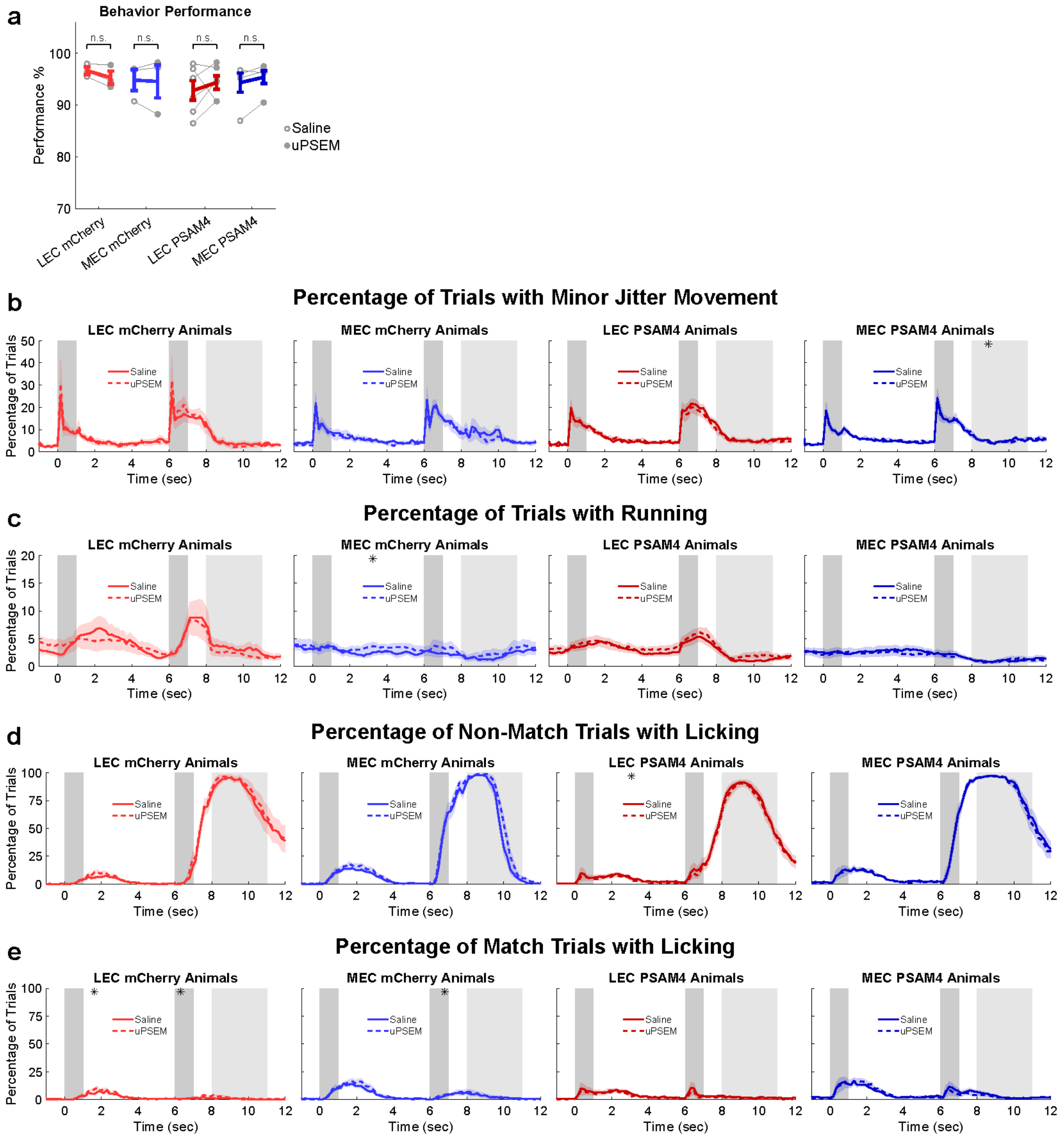

Supplementary Figure S9: LEC or MEC inhibition had no effect on behavioral performance or movements. a, Average behavioral performance was unaffected by uPSEM. Statistics are the same as similar panels in Fig. 4. Paired dots show animal averages representing the pairs of imaging days (averaging 4 recording sessions per animal per day-type; LEC mCherry = 3 animals, MEC mCherry = 3 animals, LEC PSAM4 = 6 animals, MEC PSAM4 = 5 animals). **b-e,** Minor jitter movements, running, and licking were largely unaffected by uPSEM. There were some minor significant bins, but these were random and cannot explain any other findings. Statistics are the same as similar panels in Fig 4.

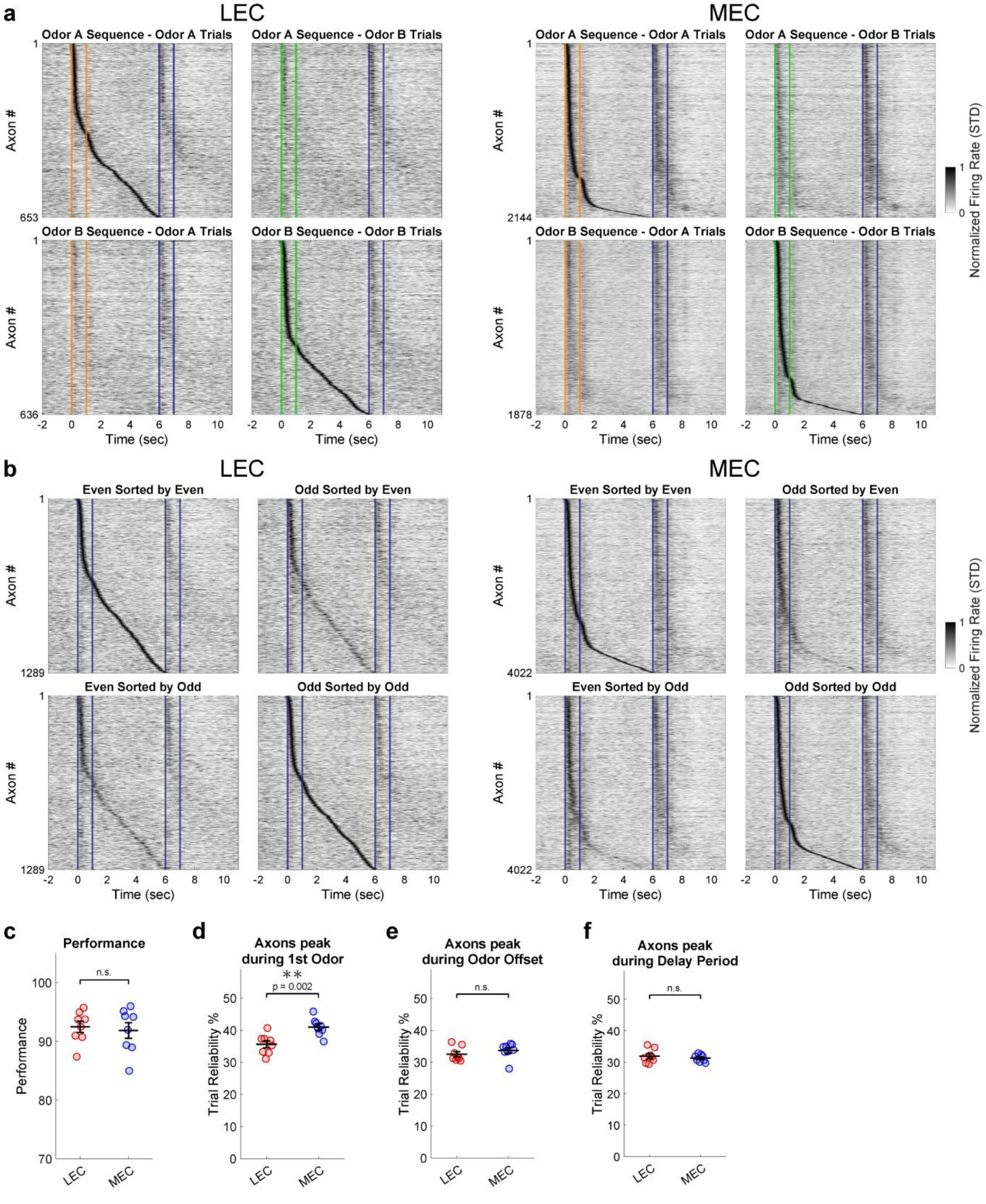

a
LEC
MEC
Odor A Sequence - Odor A Trials
Odor A Sequence - Odor B Trials
Odor B Sequence - Odor A Trials
Odor B Sequence - Odor B Trials
Axon #
653
636
2144
1878
Time (sec)
Normalized Firing Rate (STD)
b
Even Sorted by Even
Odd Sorted by Even
Even Sorted by Odd
Odd Sorted by Odd
1289
4022
c
Performance
n.s.
LEC
MEC
d
Axons peak during 1st Odor
**
p = 0.002
Trial Reliability %
e
Axons peak during Odor Offset
f
Axons peak during Delay Period

Supplementary Figure S10: LEC and MEC sequences, and 'odor axon' reliability was greater for MEC. a, LEC and MEC sequences for expert performance split by Odor A and Odor B to show the similarities and highlight odor specificity differences. **b,** LEC and MEC preferred odor sequences for expert performance doing even/odd trial validation. The robust shadows in 'even sorted by odd' and 'odd sorted by even' panels suggest that our sequence detection algorithm performed well at identifying axons with significant fields. **c** Behavioral performance was not different between LEC and MEC axon animals. Each circle represents the animal average of expert-level performance recording sessions (8 animals per group). **d-f,** Same as Fig. 5h, but for trial reliability (percentage of trials with a calcium event at its field on the preferred odor trials). Statistics are the same as Fig. 5h.

Supplementary Table 1

‘Plateau-like’ Event Rate versus Days and Performance (Linear Regression Model from Figure 3D)

Linear mixed-effects model fit by ML

Model information:

Number of observations	45
Fixed effects coefficients	4
Random effects coefficients	10
Covariance parameters	4

Formula:

Measurement ~ 1 + Days*Performance + (1 + Days | Mouse)

Model fit statistics:

AIC	BIC	LogLikelihood	Deviance
25.616	40.069	-4.808	9.6159

Fixed effects coefficients (95% CIs):

Name	Estimate	SE	tStat	DF	pValue	Lower	Upper
{'(Intercept)' }	1.3928	0.46167	3.0169	41	0.0043741	0.46043	2.3252
{'Days' }	0.076642	0.10745	0.71329	41	0.47971	-0.14036	0.29364
{'Performance' }	0.012488	0.0046261	2.6995	41	0.010042	0.0031458	0.021831
{'Days:Performance'}	-0.0013262	0.0011878	-1.1165	41	0.27071	-0.0037251	0.0010727

Random effects covariance parameters (95% CIs):

Group: Mouse (5 Levels)

Name1	Name2	Type	Estimate	Lower	Upper
{'(Intercept)'}	{'(Intercept)'}	{'std' }	0.78678	0.41489	1.492
{'Days' }	{'(Intercept)'}	{'corr'}	-0.58122	-0.93283	0.33741
{'Days' }	{'Days' }	{'std' }	0.046742	0.020791	0.10509

Group: Error

Name	Estimate	Lower	Upper
{'Res Std'}	0.19106	0.15093	0.24186

Supplementary Table 2

Success Rate of 'Plateau-like' Events during Odor versus Days and Performance (Linear Regression Model from Figure 3E)

```
Linear mixed-effects model fit by ML

Model information:
    Number of observations              45
    Fixed effects coefficients           4
    Random effects coefficients         10
    Covariance parameters                4

Formula:
    Measurement ~ 1 + Days*Performance + (1 + Days | Mouse)

Model fit statistics:
    AIC       BIC       LogLikelihood    Deviance
    255.94    270.39    -119.97          239.94

Fixed effects coefficients (95% CIs):
    Name                        Estimate    SE          tStat      DF    pValue        Lower       Upper
    {'(Intercept)'     }          27.288      5.2399     5.2077    41    5.7361e-06      16.706        37.87
    {'Days'            }         -5.7472      1.5051    -3.8186    41    0.00044614     -8.7867      -2.7077
    {'Performance'     }        -0.23909    0.076662    -3.1188    41     0.0033169    -0.39392    -0.084272
    {'Days:Performance'}        0.061483    0.017302     3.5535    41    0.00097343    0.026541     0.096426

Random effects covariance parameters (95% CIs):
Group: Mouse (5 Levels)
    Name1                  Name2                  Type            Estimate    Lower      Upper
    {'(Intercept)'}        {'(Intercept)'}        {'std' }         4.2045      1.8677    9.4652
    {'Days'       }        {'(Intercept)'}        {'corr'}             -1         NaN       NaN
    {'Days'       }        {'Days'       }        {'std' }        0.51492     0.18913    1.4019

Group: Error
    Name               Estimate    Lower     Upper
    {'Res Std'}        3.185       2.5582    3.9653
```

Supplementary Table 3

Same Odor Trials only during Odor versus Days and Performance (Linear Regression Model from Figure 3G)

Linear mixed-effects model fit by ML

Model information:

Number of observations	45
Fixed effects coefficients	4
Random effects coefficients	10
Covariance parameters	4

Formula:

Measurement ~ 1 + Days*Performance + (1 + Days | Mouse)

Model fit statistics:

AIC	BIC	LogLikelihood	Deviance
-165.16	-150.71	90.582	-181.16

Fixed effects coefficients (95% CIs):

Name	Estimate	SE	tStat	DF	pValue	Lower	Upper
{'(Intercept)' }	0.35341	0.047594	7.4255	41	4.1601e-09	0.25729	0.44952
{'Days' }	-0.04375	0.014454	-3.0268	41	0.0042587	-0.072941	-0.014559
{'Performance' }	-0.0024811	0.00064387	-3.8534	41	0.00040214	-0.0037814	-0.0011807
{'Days:Performance'}	0.00045945	0.00016039	2.8646	41	0.006555	0.00013554	0.00078336

Random effects covariance parameters (95% CIs):

Group: Mouse (5 Levels)

Name1	Name2	Type	Estimate	Lower	Upper
{'(Intercept)'}	{'(Intercept)'}	{'std' }	0.052088	0.025703	0.10556
{'Days' }	{'(Intercept)'}	{'corr'}	-0.92298	-0.99215	-0.4214
{'Days' }	{'Days' }	{'std' }	0.0068862	0.0031421	0.015092

Group: Error

Name	Estimate	Lower	Upper
{'Res Std'}	0.02662	0.021009	0.03373

Supplementary Table 4

Opposite Odor Trials only during Odor versus Days and Performance (Linear Regression Model from Figure 3H)

```
Linear mixed-effects model fit by ML

Model information:
    Number of observations              45
    Fixed effects coefficients           4
    Random effects coefficients         10
    Covariance parameters                4

Formula:
    Measurement ~ 1 + Days*Performance + (1 + Days | Mouse)

Model fit statistics:
    AIC        BIC        LogLikelihood    Deviance
    -227.68    -213.23    121.84           -243.68

Fixed effects coefficients (95% CIs):
    Name                        Estimate       SE             tStat       DF    pValue      Lower          Upper
    {'(Intercept)'     }           0.054087      0.023281      2.3233    41    0.025204     0.0070707        0.1011
    {'Days'            }        -0.0060874     0.0073586    -0.82724    41     0.41289     -0.020948     0.0087737
    {'Performance'     }       -0.00028262     0.0003574    -0.79078    41     0.43362    -0.0010044    0.00043915
    {'Days:Performance'}        7.6873e-05    8.4427e-05     0.91053    41     0.36787    -9.363e-05    0.00024738

Random effects covariance parameters (95% CIs):
Group: Mouse (5 Levels)
    Name1                  Name2                  Type            Estimate      Lower         Upper
    {'(Intercept)'}        {'(Intercept)'}        {'std' }         0.0096163     0.0041937       0.02205
    {'Days'       }        {'(Intercept)'}        {'corr'}                -1           NaN           NaN
    {'Days'       }        {'Days'       }        {'std' }        0.00016526    7.2079e-05    0.00037891

Group: Error
    Name               Estimate    Lower       Upper
    {'Res Std'}        0.014915    0.011977    0.018575
```

Supplementary Table 5

Same Odor Trials only during Delay versus Days and Performance (Linear Regression Model from Figure 3I)

Linear mixed-effects model fit by ML

Model information:

Number of observations	45
Fixed effects coefficients	4
Random effects coefficients	10
Covariance parameters	4

Formula:

Measurement ~ 1 + Days*Performance + (1 + Days | Mouse)

Model fit statistics:

AIC	BIC	LogLikelihood	Deviance
-188.95	-174.5	102.47	-204.95

Fixed effects coefficients (95% CIs):

Name	Estimate	SE	tStat	DF	pValue	Lower	Upper
{'(Intercept)' }	0.3332	0.035989	9.2584	41	1.3394e-11	0.26052	0.40589
{'Days' }	-0.06492	0.011409	-5.6903	41	1.1921e-06	-0.08796	-0.041879
{'Performance' }	-0.003252	0.00055476	-5.8621	41	6.7958e-07	-0.0043724	-0.0021317
{'Days:Performance'}	0.00072398	0.00013105	5.5247	41	2.0473e-06	0.00045933	0.00098863

Random effects covariance parameters (95% CIs):

Group: Mouse (5 Levels)

Name1	Name2	Type	Estimate	Lower	Upper
{'(Intercept)'}	{'(Intercept)'}	{'std' }	0.012591	0.0056285	0.028167
{'Days' }	{'(Intercept)'}	{'corr'}	-1	NaN	NaN
{'Days' }	{'Days' }	{'std' }	0.00011228	3.8353e-05	0.00032872

Group: Error

Name	Estimate	Lower	Upper
{'Res Std'}	0.023178	0.018617	0.028856

Supplementary Table 6

Same Odor Trials only during Odor 1 trial after – 1 trial before versus Days and Performance (Linear Regression Model from Supplemental Figure 6F)

```
Linear mixed-effects model fit by ML

Model information:
    Number of observations             45
    Fixed effects coefficients          4
    Random effects coefficients        10
    Covariance parameters               4

Formula:
    Measurement ~ 1 + Days*Performance + (1 + Days | Mouse)

Model fit statistics:
    AIC        BIC        LogLikelihood    Deviance
    -88.613    -74.159    52.306           -104.61

Fixed effects coefficients (95% CIs):
    Name                        Estimate      SE             tStat      DF    pValue        Lower         Upper
    {'(Intercept)'     }          0.70618       0.11518     6.1309    41    2.8176e-07       0.47356         0.9388
    {'Days'            }        -0.095162      0.033456    -2.8444    41     0.0069114      -0.16273      -0.027597
    {'Performance'     }       -0.0037866     0.0016737    -2.2624    41      0.029039    -0.0071668    -0.00040644
    {'Days:Performance'}         0.000915    0.00038315     2.3881    41      0.021627     0.0001412      0.0016888

Random effects covariance parameters (95% CIs):
Group: Mouse (5 Levels)
    Name1                  Name2                  Type            Estimate    Lower        Upper
    {'(Intercept)'}        {'(Intercept)'}        {'std' }        0.092239     0.041038     0.20732
    {'Days'       }        {'(Intercept)'}        {'corr'}              -1          NaN         NaN
    {'Days'       }        {'Days'       }        {'std' }        0.014773    0.0063369    0.034441

Group: Error
    Name               Estimate    Lower       Upper
    {'Res Std'}        0.069772    0.056042    0.086867
```

Supplementary Table 7

Same Odor Trials only during Delay 1 trial after – 1 trial before versus Days and Performance (Linear Regression Model from Supplemental Figure 6G)

```
Linear mixed-effects model fit by ML

Model information:
    Number of observations             45
    Fixed effects coefficients          4
    Random effects coefficients        10
    Covariance parameters               4

Formula:
    Measurement ~ 1 + Days*Performance + (1 + Days | Mouse)

Model fit statistics:
    AIC        BIC        LogLikelihood    Deviance
    -88.242    -73.788    52.121           -104.24

Fixed effects coefficients (95% CIs):
    Name                        Estimate     SE            tStat      DF    pValue        Lower         Upper
    {'(Intercept)'     }          0.8082       0.11472     7.0447    41    1.4253e-08       0.57651        1.0399
    {'Days'            }        -0.15584       0.03451    -4.5158    41    5.2415e-05      -0.22554     -0.086147
    {'Performance'     }       -0.007649     0.0017715    -4.3177    41     9.741e-05     -0.011227    -0.0040713
    {'Days:Performance'}        0.001682    0.00040261     4.1776    41    0.00015022    0.00086887     0.0024951

Random effects covariance parameters (95% CIs):
Group: Mouse (5 Levels)
    Name1                  Name2                  Type              Estimate     Lower         Upper
    {'(Intercept)'}        {'(Intercept)'}        {'std' }          0.035122     0.0044837     0.27513
    {'Days'       }        {'(Intercept)'}        {'corr'}                -1           NaN         NaN
    {'Days'       }        {'Days'       }        {'std' }         0.0043794    0.00034734    0.055217

Group: Error
    Name               Estimate    Lower       Upper
    {'Res Std'}        0.074314    0.059654    0.092575
```

Supplementary Table 8

Percentage of Odor Presentations Minor Jitter Movement versus Days and Performance (Linear Regression Model from Supplemental Figure 8B)

Linear mixed-effects model fit by ML

Model information:

Number of observations	45
Fixed effects coefficients	4
Random effects coefficients	10
Covariance parameters	4

Formula:

Measurement ~ 1 + Days*Performance + (1 + Days | Mouse)

Model fit statistics:

AIC	BIC	LogLikelihood	Deviance
327.07	341.52	-155.53	311.07

Fixed effects coefficients (95% CIs):

Name	Estimate	SE	tStat	DF	pValue	Lower	Upper
{'(Intercept)' }	18.104	11.251	1.6091	41	0.11527	-4.6181	40.826
{'Days' }	0.08597	3.3439	0.025709	41	0.97961	-6.6672	6.8391
{'Performance' }	0.10637	0.16712	0.63651	41	0.52798	-0.23113	0.44388
{'Days:Performance'}	-0.013142	0.038406	-0.34218	41	0.73396	-0.090705	0.064421

Random effects covariance parameters (95% CIs):

Group: Mouse (5 Levels)

Name1	Name2	Type	Estimate	Lower	Upper
{'(Intercept)'}	{'(Intercept)'}	{'std' }	8.1153	3.4234	19.238
{'Days' }	{'(Intercept)'}	{'corr'}	-1	NaN	NaN
{'Days' }	{'Days' }	{'std' }	0.64252	0.15147	2.7254

Group: Error

Name	Estimate	Lower	Upper
{'Res Std'}	6.9436	5.5714	8.6539

Supplementary Table 9

Percentage of Odor Presentations Running versus Days and Performance (Linear Regression Model from Supplemental Figure 8D)

```
Linear mixed-effects model fit by ML

Model information:
    Number of observations             45
    Fixed effects coefficients          4
    Random effects coefficients        10
    Covariance parameters               4

Formula:
    Measurement ~ 1 + Days*Performance + (1 + Days | Mouse)

Model fit statistics:
    AIC       BIC       LogLikelihood    Deviance
    195.19    209.64    -89.595          179.19

Fixed effects coefficients (95% CIs):
    Name                         Estimate     SE           tStat      DF    pValue     Lower        Upper
    {'(Intercept)'     }            3.887       2.3584     1.6481    41    0.10697       -0.876      8.6499
    {'Days'            }           0.8423      0.83385     1.0101    41    0.31835     -0.84169      2.5263
    {'Performance'     }         0.029725     0.036226    0.82054    41    0.41665    -0.043435     0.10288
    {'Days:Performance'}        -0.011189    0.0093042    -1.2026    41    0.23603    -0.029979    0.007601

Random effects covariance parameters (95% CIs):
Group: Mouse (5 Levels)
    Name1                  Name2                      Type            Estimate    Lower          Upper
    {'(Intercept)'}        {'(Intercept)'}        {'std' }        0.33111     9.0757e-05        1208
    {'Days'       }        {'(Intercept)'}        {'corr'}        0.19491       -0.99992     0.99996
    {'Days'       }        {'Days'       }        {'std' }         0.3248        0.13722     0.76881

Group: Error
    Name               Estimate    Lower     Upper
    {'Res Std'}        1.5166      1.1996    1.9173
```

Supplementary Table 10

Percentage of Non-Match Trials Licking During Odor versus Days and Performance (Linear Regression Model from Supplemental Figure 8F)

```
Linear mixed-effects model fit by ML

Model information:
    Number of observations              45
    Fixed effects coefficients           4
    Random effects coefficients         10
    Covariance parameters                4

Formula:
    Measurement ~ 1 + Days*Performance + (1 + Days | Mouse)

Model fit statistics:
    AIC       BIC       LogLikelihood    Deviance
    306.92    321.37    -145.46          290.92

Fixed effects coefficients (95% CIs):
    Name                        Estimate     SE           tStat       DF    pValue      Lower        Upper
    {'(Intercept)'     }           17.388      8.6153      2.0182     41    0.050141    -0.011246      34.787
    {'Days'            }            4.539      2.7938      1.6247     41     0.11189      -1.1031      10.181
    {'Performance'     }         -0.11823     0.13158    -0.89853     41     0.37415     -0.38396     0.14751
    {'Days:Performance'}        -0.041429    0.031851     -1.3007     41     0.20062     -0.10575    0.022895

Random effects covariance parameters (95% CIs):
Group: Mouse (5 Levels)
    Name1                  Name2                   Type            Estimate    Lower        Upper
    {'(Intercept)'}        {'(Intercept)'}         {'std' }         3.8285        1.2307     11.91
    {'Days'       }        {'(Intercept)'}         {'corr'}              1           NaN       NaN
    {'Days'       }        {'Days'       }         {'std' }        0.14717     0.0017134    12.641

Group: Error
    Name               Estimate    Lower     Upper
    {'Res Std'}        5.4925      4.4064    6.8465
```

Supplementary Table 11

Percentage of Non-Match Trials Licking During Odor versus Days and Performance (Linear Regression Model from Supplemental Figure 8H)

```
Linear mixed-effects model fit by ML

Model information:
    Number of observations              45
    Fixed effects coefficients           4
    Random effects coefficients         10
    Covariance parameters                4

Formula:
    Measurement ~ 1 + Days*Performance + (1 + Days | Mouse)

Model fit statistics:
    AIC       BIC       LogLikelihood    Deviance
    298.11    312.57    -141.06          282.11

Fixed effects coefficients (95% CIs):
    Name                        Estimate     SE          tStat       DF    pValue        Lower        Upper
    {'(Intercept)'     }          32.267      8.2122      3.9292     41    0.00032025      15.682       48.852
    {'Days'            }          1.7104      2.4431     0.70011     41       0.48781     -3.2235       6.6444
    {'Performance'     }        -0.32897     0.12352     -2.6634     41      0.011006    -0.57842    -0.079524
    {'Days:Performance'}       -0.014138    0.028165    -0.50197     41       0.61837   -0.071018     0.042742

Random effects covariance parameters (95% CIs):
Group: Mouse (5 Levels)
    Name1                  Name2                    Type            Estimate    Lower      Upper
    {'(Intercept)'}        {'(Intercept)'}          {'std' }         5.1998      3.1931     8.4674
    {'Days'       }        {'(Intercept)'}          {'corr'}             -1         NaN        NaN
    {'Days'       }        {'Days'       }          {'std' }        0.48551     0.34961    0.67424

Group: Error
    Name               Estimate    Lower     Upper
    {'Res Std'}        5.1371      4.1381    6.3773
```
