# Supplementary material for: Rapid formation of non-spatial hippocampal representations consistent with behavioral timescale synaptic plasticity is modulated by entorhinal input
Source: Nat Commun. 2026 Apr 10;17:5098. doi: 10.1038/s41467-026-71503-y (PMC13247236; doi:10.1038/s41467-026-71503-y)
Supplement: Supplementary file 2 — Reporting Summary [file 41467_2026_71503_MOESM2_ESM.pdf]

Corresponding author(s): Peyman Golshani
Last updated by author(s): Nov 14, 2025

Reporting Summary

Nature Portfolio wishes to improve the reproducibility of the work that we publish. This form provides structure for consistency and transparency in reporting. For further information on Nature Portfolio policies, see our Editorial Policies and the Editorial Policy Checklist.

Statistics

For all statistical analyses, confirm that the following items are present in the figure legend, table legend, main text, or Methods section.

n/a	Confirmed	
☐	☒	The exact sample size (*n*) for each experimental group/condition, given as a discrete number and unit of measurement
☐	☒	A statement on whether measurements were taken from distinct samples or whether the same sample was measured repeatedly
☐	☒	The statistical test(s) used AND whether they are one- or two-sided<br>*Only common tests should be described solely by name; describe more complex techniques in the Methods section.*
☐	☒	A description of all covariates tested
☐	☒	A description of any assumptions or corrections, such as tests of normality and adjustment for multiple comparisons
☐	☒	A full description of the statistical parameters including central tendency (e.g. means) or other basic estimates (e.g. regression coefficient) AND variation (e.g. standard deviation) or associated estimates of uncertainty (e.g. confidence intervals)
☐	☒	For null hypothesis testing, the test statistic (e.g. *F*, *t*, *r*) with confidence intervals, effect sizes, degrees of freedom and *P* value noted<br>*Give P values as exact values whenever suitable.*
☒	☐	For Bayesian analysis, information on the choice of priors and Markov chain Monte Carlo settings
☒	☐	For hierarchical and complex designs, identification of the appropriate level for tests and full reporting of outcomes
☐	☒	Estimates of effect sizes (e.g. Cohen's *d*, Pearson's *r*), indicating how they were calculated

Our web collection on statistics for biologists contains articles on many of the points above.

Software and code

Policy information about availability of computer code

Data collection	Imaging data was collected on 2 different resonant scanning two-photon microscopes (Scientifica and Bruker). Behavioral data was collected using a National Instruments data acquisition device and MATLAB (2013b, Mathworks). See Methods for additional details.
Data analysis	Imaging data was preprocessed with Suite2p (https://github.com/MouseLand/suite2p - version 0.9.2). Analysis and statistics were performed with MATLAB (2021a, Mathworks). All analysis code use for preprocessing, analysis, and making figures is available on GitHub (https://github.com/ccdorian/NonSpatialBTSP2026).

For manuscripts utilizing custom algorithms or software that are central to the research but not yet described in published literature, software must be made available to editors and reviewers. We strongly encourage code deposition in a community repository (e.g. GitHub). See the Nature Portfolio guidelines for submitting code & software for further information.

Data

Policy information about availability of data

All manuscripts must include a data availability statement. This statement should provide the following information, where applicable:

- Accession codes, unique identifiers, or web links for publicly available datasets
- A description of any restrictions on data availability
- For clinical datasets or third party data, please ensure that the statement adheres to our policy

All processed data generated in this study has been deposited in the Dryad database (https://doi.org/10.5061/dryad.573n5tbpp). Data generated for all figures and tables in this study are provided in the Source Data file.

Research involving human participants, their data, or biological material

Policy information about studies with human participants or human data. See also policy information about sex, gender (identity/presentation), and sexual orientation and race, ethnicity and racism.

Reporting on sex and gender	*Use the terms sex (biological attribute) and gender (shaped by social and cultural circumstances) carefully in order to avoid confusing both terms. Indicate if findings apply to only one sex or gender; describe whether sex and gender were considered in study design; whether sex and/or gender was determined based on self-reporting or assigned and methods used. Provide in the source data disaggregated sex and gender data, where this information has been collected, and if consent has been obtained for sharing of individual-level data; provide overall numbers in this Reporting Summary. Please state if this information has not been collected. Report sex- and gender-based analyses where performed, justify reasons for lack of sex- and gender-based analysis.*
Reporting on race, ethnicity, or other socially relevant groupings	*Please specify the socially constructed or socially relevant categorization variable(s) used in your manuscript and explain why they were used. Please note that such variables should not be used as proxies for other socially constructed/relevant variables (for example, race or ethnicity should not be used as a proxy for socioeconomic status). Provide clear definitions of the relevant terms used, how they were provided (by the participants/respondents, the researchers, or third parties), and the method(s) used to classify people into the different categories (e.g. self-report, census or administrative data, social media data, etc.) Please provide details about how you controlled for confounding variables in your analyses.*
Population characteristics	*Describe the covariate-relevant population characteristics of the human research participants (e.g. age, genotypic information, past and current diagnosis and treatment categories). If you filled out the behavioural & social sciences study design questions and have nothing to add here, write "See above."*
Recruitment	*Describe how participants were recruited. Outline any potential self-selection bias or other biases that may be present and how these are likely to impact results.*
Ethics oversight	*Identify the organization(s) that approved the study protocol.*

Note that full information on the approval of the study protocol must also be provided in the manuscript.

Field-specific reporting

Please select the one below that is the best fit for your research. If you are not sure, read the appropriate sections before making your selection.

- [x] Life sciences
- [ ] Behavioural & social sciences
- [ ] Ecological, evolutionary & environmental sciences

For a reference copy of the document with all sections, see nature.com/documents/nr-reporting-summary-flat.pdf

Life sciences study design

All studies must disclose on these points even when the disclosure is negative.

Sample size	Group sizes were chosen to minimize the number of animals while being sufficient for consistency of the observed effects. This conforms to ethical standards and is comparable to other similar studies (Bellafard et al. 2024 Nature, Grienberger and Magee 2022 Nature, Li et al. Nature Neuroscience 2017).
Data exclusions	As described in the methods, mice were excluded after post-hoc histology showing insufficient viral expression or expression outside of the target region. Additionally, for axon imaging experiments, sessions with behavioral performance below 85% (expert-level) and/or imaging movies with failed motion registration in Suite2p were excluded.
Replication	All findings were replicated across animals and days. See results and methods for exact details of numbers of mice, days, and trials for reach experiment.
Randomization	Animals were assigned groups before surgery pseudorandomly to also control for the covariates of sex and group housing. Trials types were pseudorandomly delivered to ensure the delivery of the same total number of each trial type.
Blinding	When possible, the individual experimenter was blinded to the group of each animal during data collection. Blinding to the drug delivery of saline or uPSEM was no possible because drugs were prepared immediately before injections; however, all mice underwent identical conditions and protocols. Blinding was not necessary during analysis as everything was automated with identical parameters.

Reporting for specific materials, systems and methods

We require information from authors about some types of materials, experimental systems and methods used in many studies. Here, indicate whether each material, system or method listed is relevant to your study. If you are not sure if a list item applies to your research, read the appropriate section before selecting a response.

Materials & experimental systems

n/a	Involved in the study
☒	☐ Antibodies
☒	☐ Eukaryotic cell lines
☒	☐ Palaeontology and archaeology
☐	☒ Animals and other organisms
☒	☐ Clinical data
☒	☐ Dual use research of concern
☒	☐ Plants

Methods

n/a	Involved in the study
☒	☐ ChIP-seq
☒	☐ Flow cytometry
☒	☐ MRI-based neuroimaging

Animals and other research organisms

Policy information about studies involving animals; ARRIVE guidelines recommended for reporting animal research, and Sex and Gender in Research

Laboratory animals

A total of 9 adult male and 8 female mice (8-16 weeks old, C57BL/6J The Jackson Laboratory 000664) were used for simultaneous in vivo calcium CA1 imaging and EC chemogenetic experiments (Figures 1 and 4). These mice were divided into 4 groups: LEC mCherry n=3, MEC mCherry n=3, LEC PSAM4 n=6, MEC PSAM4 n=5. A total of 3 adult female mice (25-35 weeks old) were used for simultaneous in vivo calcium CA1 imaging and holographic optogenetic stimulation experiments (Figure 2). These 3 positive offspring were chosen from a breeding of B6;DBA-Tg(tetO-GCaMP6s)2Niell/J (The Jackson Laboratory 024742) or B6;D2-Tg(tetO-GCaMP8s)1Genie/J (The Jackson Laboratory 037717) crossed with B6.Cg-Tg(Camk2a-tTA)1Mmay/DboJ (The Jackson Laboratory 007004). A total of 2 adult male and 3 female mice (8-16 weeks old, C57BL/6J The Jackson Laboratory 000664) were used for in vivo calcium CA1 imaging during learning experiments (Figure 3). A total of 7 adult male and 9 female mice (8-16 weeks old, C57BL/6J The Jackson Laboratory 000664) were used for in vivo calcium EC axon imaging experiments (Figure 5). These mice were divided into 2 groups: LEC n=8, MEC n=8. All were experimentally naïve and housed in the vivarium under a 12-hour light/dark cycle. All mice were group housed (2-4 per cage) except for 2 that had to be separated following surgery because of fighting.

Wild animals

No wild animals were used in this study.

Reporting on sex

Sexes were balanced in the study design. Sex-based analysis was not performed because we do not expect to find sex differences, and the limited number of animals does not provide enough power to yield statistically significant differences.

Field-collected samples

No field-collected samples were used in this study.

Ethics oversight

All of the experiments were conducted according to the National Institute of Health (NIH) guidelines and with the approval of the Chancellor's Animal Research Committee of the University of California, Los Angeles.

Note that full information on the approval of the study protocol must also be provided in the manuscript.

Plants

Seed stocks

Report on the source of all seed stocks or other plant material used. If applicable, state the seed stock centre and catalogue number. If plant specimens were collected from the field, describe the collection location, date and sampling procedures.

Novel plant genotypes

Describe the methods by which all novel plant genotypes were produced. This includes those generated by transgenic approaches, gene editing, chemical/radiation-based mutagenesis and hybridization. For transgenic lines, describe the transformation method, the number of independent lines analyzed and the generation upon which experiments were performed. For gene-edited lines, describe the editor used, the endogenous sequence targeted for editing, the targeting guide RNA sequence (if applicable) and how the editor was applied.

Authentication

Describe any authentication procedures for each seed stock used or novel genotype generated. Describe any experiments used to assess the effect of a mutation and, where applicable, how potential secondary effects (e.g. second site T-DNA insertions, mosiacism, off-target gene editing) were examined.
