# Supplementary material for: Rapid formation of non-spatial hippocampal representations consistent with behavioral timescale synaptic plasticity is modulated by entorhinal input
Source: Nat Commun. 2026 Apr 10;17:5098. doi: 10.1038/s41467-026-71503-y (PMC13247236; doi:10.1038/s41467-026-71503-y)
Supplement: Supplementary file 3 — Transparent Peer Review file [file 41467_2026_71503_MOESM3_ESM.pdf]

nature portfolio

Peer Review File

Rapid formation of non-spatial hippocampal representations consistent with behavioral timescale synaptic plasticity is modulated by entorhinal input

Corresponding Author: Dr Peyman Golshani

This file contains all reviewer reports in order by version, followed by all author rebuttals in order by version.

Version 0:

Reviewer comments:

Reviewer #1

(Remarks to the Author)
Summary
The manuscript Behavioral timescale synaptic plasticity in the hippocampus creates non-spatial representations during learning and is modulated by entorhinal inputs by Dorian et al. proposes that large, rare "plateau-like" somatic calcium transients in CA1—detected as ≥10 SD ΔF/F events with minimal preceding activity—drive the rapid formation of odor-selective firing fields during a two-odor head-fixed DNMS working-memory task. Chemogenetic silencing of MEC is reported to reduce event frequency, while LEC silencing lowers the probability that an event yields a field and impairs odor decoding. Single-cell holographic stimulation of ChRmine is used to "write" odor fields on demand. The authors conclude that behavioral-timescale synaptic plasticity (BTSP) underlies non-spatial odor learning and that MEC and LEC provide dissociable "teaching" and "content" signals.
The study elegantly employs modern optical tools to explore non-spatial BTSP, but several fundamental issues currently undermine its central conclusions. First, the authors claim to investigate dendritic plateau potentials yet rely exclusively on slow somatic calcium imaging—equating asymmetric somatic ΔF/F transients with true dendritic plateau events without any direct tuft recordings or voltage measurements. Second, their single-cell "causation" paradigm uses a Kv2.1-targeted, non-selective ionic-conductance opsin delivered to the soma and proximal dendrite; driving strong proximal depolarization and large somatic calcium signals after tens of holographic pulses does not demonstrate integration at distal dendrites or genuine BTSP mechanisms. Third, the use of a delayed non-match-to-sample working-memory task conflates one-shot novelty learning with sustained maintenance, decision, and reward processes—yet analyses focus mainly on expert performance rather than the critical first exposures. Fourth, despite distinct sample versus test epochs, delay periods, and reward contexts, all odor presentations are pooled, masking potentially divergent network and neuromodulatory states. Fifth, only two fixed odors in a rigid DNMS structure are tested, preventing assessment of BTSP's role in richer, more flexible olfactory learning or generalization. Finally, methodological and statistical weaknesses—including an asymmetric shuffle null, liberal uncorrected thresholds, selective event filtering, small sample sizes/large variance for critical induction experiments, and seemingly false errorbars, eg, Fig. 4c/g—as well as the complete omission of oscillatory-phase considerations, further erode confidence in the claimed mechanism. This is accompanied by a reported lack of any correlating effect on the relation to behavior.
A thorough reanalysis that validates genuine dendritic plateau detection and tightens statistical controls—and that tempers causal language or adds methodologically suitable experiments to control that—is required before the manuscript can be considered for publication at Nat. comms.

Major Points
Task choice versus claimed mechanism
Using a delayed non-match-to-sample working-memory task conflates encoding, maintenance, decision and reward processes, yet the key novelty claim hinges on "one-shot" learning. The analysis centers on expert performance rather than the critical initial exposures. A simpler multi-odor novelty paradigm would help to support the claim and a critical discussion of the lack a behavioral correlation would be helpful; alternatively the framing must shift to steady-state experience dependence rather than one-shot encoding.

Pooling of sample and test presentations

Combining encoding (sample) and retrieval/decision (test) odor epochs ignores distinct neuromodulatory and network states, potentially conflating mechanisms that differ between presentation positions. Separate analyses of event frequency, amplitude, success rate and field selectivity for sample versus test are required—and pooling justified only if no significant differences exist.

Unvalidated somatic proxy for dendritic plateaus
Equating somatic ΔF/F transients ≥10 SD with dendritic plateau potentials is unsubstantiated. Electrotonic filtering blurs and attenuates distal events, and high-amplitude somatic bursts can masquerade as plateaus. The authors should add direct measurements to support the claim of dendritic integration and ideally provide tuft imaging or voltage recordings—or explicitly temper mechanistic claims.

Incomplete reporting of event–field contingencies
Key denominators are missing: the number of large events that do not produce fields and the number of fields arising without a detected event. Without a full contingency analysis and permutation or time-shift controls, the strength and specificity of the event→field link cannot be assessed objectively.

Circular shuffle null mismatch and liberal thresholds for event detection
The “circular shuffle” null appears to include all odor windows (varying multiple odor trials depending where in the trial number the event appeared (larger than 10, smaller than 90)—even those filtered out of the real data—which violates exchangeability and likely biases p-values. Coupled with an uncorrected 90th-percentile threshold (allowing up to 10% false positives per test) and no multiple-comparison control, the field-significance method risks substantial type I error. A matched filtered null, FDR correction, and simulation-based false positive rates are required.

Selective pre-exclusion of recent activity
Requiring no activity ±2 s in the three preceding odor presentations excludes neurons with ramping or overlapping events, biasing the sample toward “clean” silent-to-active transitions. This pre-selection may inflate apparent specificity and overlook biologically relevant induction contexts. The impact of this filter must be quantified and, if necessary, replaced with a continuous covariate rather than binary exclusion.

Holographic induction paradigm limitations
Single-cell optogenetic stimulation results are shown for very few neurons and mice, with no clear reporting of total stimulation attempts or failure rates. The use of Kv2.1-targeted ChRmine biases activation to proximal compartments, likely differing from natural tuft-driven plateaus. Negative controls (sub-threshold, off-epoch, opsin-negative) and direct comparison of evoked versus spontaneous event waveforms are essential before claiming sufficiency for field writing.

Absence of oscillatory-phase context
BTSP and dendritic excitability are known to be gated by theta phase. Without LFP recordings or phase analysis, the role of rhythmic coordination is speculative. The authors should either provide phase-locking data or explicitly limit mechanistic conclusions.

Minor Points
• Limited odor set: Only two odors are tested. Generalization to richer odor spaces requires additional stimuli or tempered scope.
• Temporal window choice: The use of a fixed 6 s window and occasional 1 s pre-event bins is never justified; analyses should demonstrate robustness to different window lengths.
• Anatomical sampling: CA1 exhibits proximodistal and radial innervation gradients from LEC versus MEC; imaging coordinates should be reported and included as covariates.
• Potentially wrong depiction of errorbars: carefully check errorbar sizes in the figures, most obviously in Fig4c/g; large variance groups seem to have identical or smaller errorbars compared to low variance groups, despite comparable sample size.
• Terminology clarity: Clear operational definitions for “odor field,” “odor cell,” “plateau-like event” and “success rate” should be provided in Methods.
• Causal language: Phrases such as “plateaus drive fields” should be replaced with “precede” or “are associated with” unless necessity is demonstrated.
• Figure legends: Denote whether n refers to neurons, events or mice; show per-mouse data points where possible.
• Threshold justification: Percentile cutoffs for induction and field detection (90th/95th) must be rationalized or chosen via ROC/FDR frameworks.

(Remarks on code availability)

Reviewer #2

(Remarks to the Author)
The study by Dorian et al. investigates whether behavioral timescale plasticity (BTSP) can drive the formation of memory-related non-spatial hippocampal representations. This question is addressed by performing calcium imaging from hippocampal CA1 cells as well as from LEC and MEC axons in CA1 while mice are performing an olfactory working memory task. In addition, optogenetic induction of BTSPs in CA1 and chemogenetic manipulations of LEC and MEC are performed

to establish that BTSPs are causal in generating odor-related responses and that LEC and MEC are contributing to the success and frequency of BTSPs. Furthermore, recordings during task acquisition show that the relative frequency of BTSP is increased early in training on the odor task. The series of experiments is performed with high rigor, and appropriate statistical methods are used. Importantly, the experiments yield a highly novel result, which is that BTSP is a general plasticity mechanism and not limited to the formation of spatial fields. In addition to reporting this key result, the study also characterizes some mechanism of how BTSPs contribute to the encoding of non-spatial representations. As with any novel phenomenon, the study of course raises a number of interesting questions for future work, such as the limited effectiveness of BTSPs to generate fields later in the delay. The study is nonetheless comprehensive as well as exceptional in its impact and novelty. There are only a few minor points that, if addressed, would further improve the manuscript.
Minor points:
(1) The sentence in the abstract 'Using holographic stimulation … ' is somewhat imprecise because spontaneously-occurring events do not need stimulation.
(2) Fig. 1c. The blue bars are too small to be legible.
(3) Panels in Fig. 1g, S1 and S2 seem to show BTSP events later in the delay periods that do not result in fields. Although the low effectiveness of these events to result in fields is pointed out in the text, this could also be more explicitly labelled in the figure panels and mentioned in the figure legends.
(4) Fig. 1i and S3. Although the results that small events rarely form odor fields is clear, it is less obvious why the timing of the small events shows a value around zero distance from the reference field. If these were completely random events there should at the minimum be an increase variance because the event time and field time are unrelated. Is it possible that 'small events' correspond to an odor fields that occur at a low rate, but nonetheless repeatedly around the same time? Perhaps this point is so obvious that it is not explicitly mentioned. It would nonetheless be helpful for non-experts to provide some explanation for the zero distance other than referring to randomness.
(5) Fig. 3 d, e, g and i, Fig. S4f and g, Fig. S6b, d, f and h. All the correlations with performance % (or the lack thereof) seem to be a somewhat trivial consequence of effects occurring (or not occurring) early in training. Performance is obviously lower early in training, and any effect that varies with training days would thus necessarily also show a correlation with performance. If the question is asked whether performance is a key predictor in addition to training days, one would have to do much more sophisticated analysis than a simple regression. The relative effect of these two variables (training day and performance) would need to be dissociated, for example by a partial correlation or an analysis of variance that considers the relative contribution of each of the two factors (training day and performance). Appropriate statistical methods should therefore be used, or all of the panels plotting a variable against performance % should be removed along with the corresponding text.
(6) The use of the term 'odor axon' for MEC axons can be somewhat misleading. While the activity of these axons is clearly timed with regard to stimulus onset and offset, few have selectivity for a particular odor. For comparison, we would not use the term 'place cell' if a cell were not selective for a particular place.
(7) The very last phrase in the Results section 'being relayed indirectly through the CA1 perforant path as well' does not sound correct. First, the sentence seems to focus on LEC inputs, but the perforant path through DG and CA3 would be conveying merged MEC and LEC inputs. Second, there is no perforant path with a direct connection to CA1 and the meaning of a 'CA1 perforant path' is therefore not evident.
(8) In the Results and in the Discussion, it would be more precise to distinguish two potential types of effects as a consequence of manipulating entorhinal inputs to CA1. One type is plasticity/BTSP-related, and although detectable in well-trained mice, may no longer be as behaviorally relevant as it would be if the manipulation were done earlier in training. The second type are effects on hippocampal activity patterns as a consequence of loss of information from LEC or MEC. With any manipulation of a brain region, effects on plasticity and from loss of inputs to connected circuits can often not be fully dissociated. It is nonetheless important to conceptually distinguish between these types of effects and to acknowledge that both contribute to differences in neuronal firing patterns during/after manipulations.

Stefan Leutgeb

(Remarks on code availability)
I have reviewed the files and all details for running the code seems to be provided. However, I do not have the resources to run the code and did not install it.

The authors refer to large data files in their code. It should perhaps be more explicitly states whether the data files will be uploaded to a repository or are available upon request.

Version 1:

Reviewer comments:

Reviewer #1

(Remarks to the Author)
Thank you for the detailed rebuttal and substantial revisions. Several of my earlier concerns are now at least partially addressed, especially the change in terminology to "plateau-like" events, the added discussion of somatic imaging caveats, and the corrections to error bars. However, I still feel that the strength and scope of the main claims exceed what the data can support, and I would strongly encourage further tempering of the mechanistic and causal language, as well as clearer acknowledgment of alternative interpretations.

Mechanistic and causal claims
While the authors have removed some explicit causal phrases and now refer to “plateau-like” rather than “BTSP-like” events, the title, abstract, and discussion still largely present the results as demonstrating that BTSP is a general plasticity mechanism for non-spatial representations. In particular, the concluding statement in the abstract that the findings “demonstrate a causal role of BTSP” is not supported by the present data. Given that all inferences are based on somatic calcium signals and an operational definition of ≥10 SD events, this remains too strong. The same somatic signature could arise from different underlying mechanisms (e.g. bursts, mixed dendritic/somatic events), and the data demonstrate correlation and temporal precedence rather than necessity or sufficiency. I would recommend consistently rephrasing to something more neutral, such as “rare, large somatic calcium events consistent with BTSP models can precede the formation of some odor fields in this task,” and avoiding language that implies that BTSP per se has been established as the mechanism.

Operational definition and circularity
The revised text better documents numerators/denominators and threshold choices, but the core concern about circularity remains. “Possible induction events” are defined using stringent criteria (low prior activity, no strong field in the preceding trials, restricted trial window), and “success” is then defined as a new, reliable field emerging near the event in time. This effectively preselects events in which a silent-to-active transition occurs with the desired temporal asymmetry and discards cases where fields emerge more gradually, overlap existing activity, or arise without a qualifying event. The binary analysis therefore builds the BTSP-like pattern into the inclusion criteria and will, by construction, emphasize one-shot, asymmetric field formation. The more continuous “threshold-free” analysis is a welcome addition, but the manuscript should more clearly acknowledge that the binary/“induction” analysis is conditional on these strong filters and cannot be taken as an unbiased estimate of how often plateau-like events genuinely drive fields versus other scenarios.

Generality and time representations
The manuscript still suggests that BTSP underlies non-spatial representations more broadly, including time. However, the data show that only a very small minority of plateau-like events occurring after odor offset precede time fields, and optogenetic induction in the delay period did not reliably generate fields. The discussion now notes that delay-related effects are weaker and rare, but the claims about generalization to time and “internally generated” codes still read as if they are an established extension of the mechanism rather than an open question. I would recommend explicitly stating that the present data provide only limited support for BTSP-like contributions to time fields and that the role of such events in temporal coding remains unresolved.

Behavioral relevance and EC silencing
The chemogenetic manipulations clearly modulate plateau-like event rates, success probabilities, and odor decoding in CA1, but have no detectable effect on behavioral performance in the expert animals. This is now documented more clearly, but the manuscript still leans toward a causal interpretation in which BTSP-dependent representations are central for task solution. Given the lack of behavioral effect in the regime actually recorded, a more balanced formulation would be that LEC and MEC manipulations alter neural representations and event statistics during expert performance, but their necessity for behavior under these conditions is not demonstrated. Any stronger inference about “BTSP creating behaviorally relevant representations” should be softened accordingly or explicitly framed as a hypothesis for future work (e.g. during acquisition or more demanding variants of the task).

Statistical issues
- The authors state that they now use linear regression to separate day and performance, and they have corrected the chemogenetic error bars, which is appreciated. Nonetheless, several statistical concerns remain:
- Regression analyses treat each animal–day as an independent data point without modeling mouse as a random effect. With only five mice followed over multiple days, this violates the hierarchical structure of the data and likely underestimates uncertainty on day/performance effects.
Event-level analyses still appear to treat individual events as independent observations in some tests, even though many events come from the same cells and animals, again inflating effective n and p-values.
- The multi-factor ANOVAs in the chemogenetic experiments are based on very small group sizes (e.g. n = 3 controls), where parametric assumptions are fragile and effect sizes or confidence intervals would be more informative than marginal p-values.

These points do not overturn the qualitative trends, but they further argue for conservative statements about effect robustness and for avoiding strong mechanistic or causal claims based on borderline statistics.

Context from Sumegi et al.
The authors now cite Sumegi et al. as using somatic calcium to study BTSP-like phenomena. Importantly, that work also shows that large somatic Ca^{2+} events with BTSP-like signatures are neither necessary nor sufficient for place field formation and that diverse calcium dynamics can underlie new fields. This does not contradict the present study, but it reinforces the idea that large somatic “plateau-like” events are one of several possible routes to field formation. In my view, this makes it even more important that the current manuscript frame its main conclusion as the identification of one correlated pathway in a specific non-spatial task, rather than as establishing BTSP as the general mechanism for non-spatial representations.

In summary, the additional analyses and revisions improve the manuscript, but I still recommend a further toning down of mechanistic and causal language, more explicit discussion of alternative mechanisms and the limitations of the operational definition, clearer acknowledgment of the lack of behavioral impact of EC silencing under the conditions tested, and a more cautious treatment of generalization to time fields.

(Remarks on code availability)

Reviewer #2

(Remarks to the Author)
All of the previously raised points have been thoroughly addressed.

(Remarks on code availability)

Version 2:

Reviewer comments:

Reviewer #1

(Remarks to the Author)
The authors have addressed all of my points. Congratulations to the authors on the thorough revisions. I recommend acceptance for publication.

(Remarks on code availability)

Open Access This Peer Review File is licensed under a Creative Commons Attribution 4.0 International License, which permits use, sharing, adaptation, distribution and reproduction in any medium or format, as long as you give appropriate credit to the original author(s) and the source, provide a link to the Creative Commons license, and indicate if changes were made.
In cases where reviewers are anonymous, credit should be given to 'Anonymous Referee' and the source.
The images or other third party material in this Peer Review File are included in the article’s Creative Commons license, unless indicated otherwise in a credit line to the material. If material is not included in the article’s Creative Commons license and your intended use is not permitted by statutory regulation or exceeds the permitted use, you will need to obtain permission directly from the copyright holder.
To view a copy of this license, visit https://creativecommons.org/licenses/by/4.0/

Response to Reviewers:

We thank the reviewers for their careful consideration of our manuscript. The reviewers' comments and suggestions led us to substantially improve the manuscript. We are grateful that the reviewers felt that our "comprehensive" study was performed "elegantly", and that the experiments and results were "highly novel". Yet, the reviewers had some concerns that required us to perform further analyses. These new analyses have further strengthened our conclusions, and we have made appropriate changes to temper mechanistic claims. We have addressed all the points raised by the reviewers and outline our response below.

Reviewer #1: Summary:
The manuscript Behavioral timescale synaptic plasticity in the hippocampus creates non-spatial representations during learning and is modulated by entorhinal inputs by Dorian et al. proposes that large, rare "plateau-like" somatic calcium transients in CA1—detected as ≥10 SD ΔF/F events with minimal preceding activity—drive the rapid formation of odor-selective firing fields during a two-odor head-fixed DNMS working-memory task. Chemogenetic silencing of MEC is reported to reduce event frequency, while LEC silencing lowers the probability that an event yields a field and impairs odor decoding. Single-cell holographic stimulation of ChRmine is used to "write" odor fields on demand. The authors conclude that behavioral-timescale synaptic plasticity (BTSP) underlies non-spatial odor learning and that MEC and LEC provide dissociable "teaching" and "content" signals. The study elegantly employs modern optical tools to explore non-spatial BTSP, but several fundamental issues currently undermine its central conclusions. First, the authors claim to investigate dendritic plateau potentials yet rely exclusively on slow somatic calcium imaging—equating asymmetric somatic ΔF/F transients with true dendritic plateau events without any direct tuft recordings or voltage measurements. Second, their single-cell "causation" paradigm uses a Kv2.1-targeted, non-selective ionic-conductance opsin delivered to the soma and proximal dendrite; driving strong proximal depolarization and large somatic calcium signals after tens of holographic pulses does not demonstrate integration at distal dendrites or genuine BTSP mechanisms. Third, the use of a delayed non-match-to-sample working-memory task conflates one-shot novelty learning with sustained maintenance, decision, and reward processes—yet analyses focus mainly on expert performance rather than the critical first exposures. Fourth, despite distinct sample versus test epochs, delay periods, and reward contexts, all odor presentations are pooled, masking potentially divergent network and neuromodulatory states. Fifth, only two fixed odors in a rigid DNMS structure are tested, preventing assessment of BTSP's role in richer, more flexible olfactory learning or generalization. Finally, methodological and statistical weaknesses—including an asymmetric shuffle null, liberal uncorrected thresholds, selective event filtering, small sample sizes/large variance for critical induction experiments, and seemingly false errorbars, eg, Fig. 4c/g—as well as the complete omission of oscillatory-phase considerations, further erode confidence in the claimed mechanism. This is accompanied by a reported lack of any correlating effect on the relation to behavior. A thorough reanalysis that validates genuine dendritic plateau detection and tightens statistical controls—and that tempers causal language or adds methodologically suitable experiments to control that—is required before the manuscript can be considered for publication at Nat. comms.

We thank the reviewer for their detailed and thoughtful feedback. We have addressed all the analytical concerns, expanded discussions of caveats and complexity, and tempered some of the causal language. With these revisions, we believe the manuscript is much stronger and precise with its novelty. We address all these issues in a point-by-point discussion below.

Reviewer #1: Major Points:

Task choice versus claimed mechanism

The key novelty claim hinges on "one-shot" learning. The analysis centers on expert performance rather than the critical initial exposures. A simpler multi-odor novelty paradigm would help to support the claim and a critical discussion of the lack a behavioral correlation would be helpful; alternatively the framing must shift to steady-state experience dependence rather than one-shot encoding.

We agree with the reviewer that the increased rate of BTSP-like events primarily on the first day of learning suggests that this rate increase may be largely driven by novelty. We have removed mentions of "one-shot learning" as it pertains to our findings. Additionally, we argue that the primary novelty of our findings is the discovery of these BTSP-like events in a non-spatial task. Our experiments were not designed to investigate the plasticity mechanism of BTSP itself, but rather to characterize the phenomenon in a new context of non-spatial representations during expert performance, which suggests that BTSP may underly the formation of other hippocampal representations beyond place cells.

To disentangle the contribution of novelty and performance, we have reanalyzed the relevant data. Rather than performing a simple correlation between rates of BTSP-like events and performance, we now use a linear regression model that isolates the effect of day or performance on the measure. We now display the t- and p-values associated with only the effect of performance for all scatter plots (Figure 3d,e,g,h,I, Supplementary Figure S4f,g, and Supplementary Figure S6b,d,f,h). Additionally, we include the results of each linear regression model in Supplementary Tables 1-11 to demonstrate the relative effect of day, performance, and the interaction between the two factors. Although there are changes from the originally displayed p-values, our overall conclusions about the combined effect of day and performance are unaffected. We also include a more thorough discussion of the new statistical approach and results.

Pooling of sample and test presentations

Combining encoding (sample) and retrieval/decision (test) odor epochs ignores distinct neuromodulatory and network states, potentially conflating mechanisms that differ between presentation positions. Separate analyses of event frequency, amplitude, success rate and field selectivity for sample versus test are required—and pooling justified only if no significant differences exist.

This is an important point as the first (sample) and second (test) odors could be encoded differently in our CA1 neurons. We have added Supplementary Figure S4 (also included below) that compares results when restricting analysis to either only the first or second odor. We find no difference, which aligns with previous work in our lab studying odor representations in CA1 during the same task (Figure S2 in (Taxidis et al., 2020)).

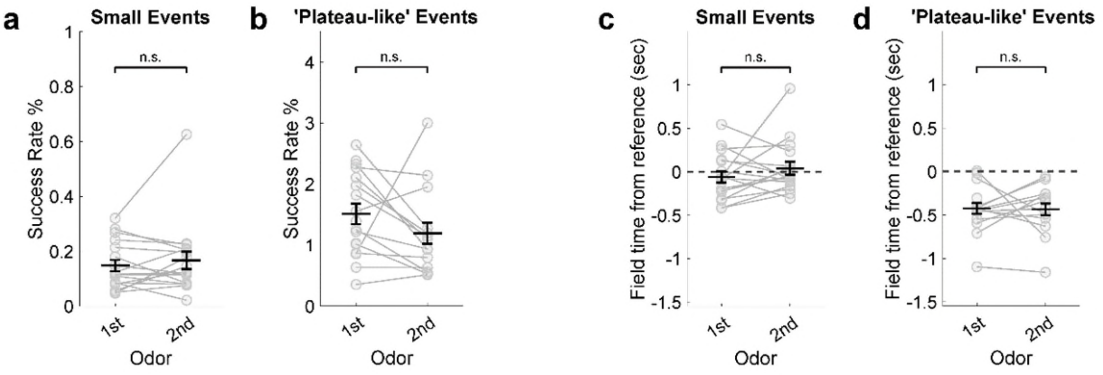

Supplementary Figure S4: 'BTSP-like' events are similar for first and second odor presentation periods. Detection analysis was re-run using either the first odors or second odor presentations. Complementing previous work in the lab using the same task[19], we find no difference between the two odors. **a,** Success rates of small events are not different between first and second odors (paired t-test, p = 0.4342). Dots and lines represent each animal (17 animals, which are the average for each of their 8 recording sessions). Error bars represent the mean and SEM. **b,** Same as (a), but for 'plateau-like' events, which are also not different (paired t-test, p = 0.2303). **c,** Similar to (a), but calculating the field time from reference as in Figure 1i for all newly formed odor-fields (paired t-test, p = 0.2429). **d,** same as (c), but for 'plateau-like' events (paired t-test, p = 0.7371).

Unvalidated somatic proxy for dendritic plateaus

Equating somatic ΔF/F transients ≥10 SD with dendritic plateau potentials is unsubstantiated. Electrotonic filtering blurs and attenuates distal events, and high-amplitude somatic bursts can masquerade as plateaus. The authors should add direct measurements to support the claim of dendritic integration and ideally provide tuft imaging or voltage recordings—or explicitly temper mechanistic claims.

While our calcium imaging approach has some limitations, the restriction to somatic events is not unique, and it aligns with previous spatial BTSP research that recorded 'BTSP-like' events using somatic calcium imaging (Grienberger & Magee, 2022; Priestley et al., 2022; Sumegi et al., 2025). The standard for measuring BTSP in place cell formation has been performing intracellular recordings from somata and the main induction mechanism has been injecting current into the soma. In Grienberger & Magee, 2022, a threshold of 3STD is used for CA1 place cell detection. Our use of 10STD is extremely conservative and limits analysis to only the very largest calcium somatic events that likely propagate through most dendrites to many synapses (Milstein et al., 2021; Takahashi & Magee, 2009). Furthermore, characterization of the BTSP-like events in Figure 1 is displayed across a range of threshold values to demonstrate the relationship between event amplitude and odor field measurements.

To address the reviewer's point, we now refer to calcium events larger than 10STD to be 'plateau-like' events, and we have tempered mechanistic claims throughout the manuscript by removing causal language. For example, we have replaced phrases such as "induction events drive the formation of odor-fields" with "*induction events precede the formation of odor-fields*". In the Discussion, we also now discuss the challenge of understanding a theorized dendritic mechanism by recording somatic events:

"*Our somatic calcium imaging approach has the advantage of recording many neurons to find rare 'BTSP-like' events, but experiments to investigate dendritic dynamics will be valuable for further understanding the underlying mechanisms of CA3 and EC inputs in driving BTSP. By focusing our analysis to only the largest calcium events, we likely isolate events that propagate through most dendrites to many synapses, but we recognize that some of these events may be large somatic spiking bursts rather than those originating from dendritic plateau-potentials. Voltage and calcium recordings of dendritic tufts at different depths with the stratum radiatum and stratum lacunosum-moleculare will be necessary to evaluate the contributions of CA3 and EC inputs to the CA1 neurons*."

Incomplete reporting of event–field contingencies

Key denominators are missing: the number of large events that do not produce fields and the number of fields arising without a detected event. Without a full contingency analysis and permutation or time-shift controls, the strength and specificity of the event→field link cannot be assessed objectively.

We apologize for the omission. In addition to success rates, we now list numerators and denominators for key findings. We have clarified the text in the results section. We now state:

"*We thus considered a reference calcium event as 'plateau-like' when its amplitude was > 10 STD. Plateau-like events were rare (122,605 out of 16,215,467 total events recorded = 0.76%) and matched the shapes and amplitudes of previously described somatic calcium plateau events (Supp. Fig. S3a-d). Approximately 8% of plateau like events that occurred during the odor cue or within the ensuing 0.5 seconds preceded a subsequent odor-field formation (294 'successes' out of 3876 total events). This increased to ~15% during the second half of the odor presentation cue (Fig. 1j). Only 26 'plateau-like' events beyond 0.5 seconds after odor offset preceded a subsequent time-field formation (8% of the 320 successful events). The odor-fields preceded by plateau-like events peaked 0.42 ± 0.14 seconds earlier in trial-time compared to the reference event (Fig. 1k). In contrast, only 0.28% "small events" (<10 STD amplitude) during the odor cue or the ensuing 0.5 seconds (254 out of 91,635 total events) preceded new odor fields (Supp. Fig. S5a), These events represent the noise floor of our detection criteria and the rare odor-field formation that formed without a preceding 'plateau-like' event. These rare fields were symmetrically distributed around the reference event timepoint with increased variance (Supp. Fig. S5b). We thus label 'plateau-like' events preceding an odor-field as 'BTSP-like'. We observed no difference in success rates or asymmetrical field-formation between first and second odor (Supp. Fig. S4). Therefore, all odor cues were pooled for further analysis.*"

Circular shuffle null mismatch and liberal thresholds for event detection

The "circular shuffle" null appears to include all odor windows (varying multiple odor trials depending where in the trial number the event appeared (larger than 10, smaller than 90)—even those filtered out of the real data—which violates exchangeability and likely biases p-values. Coupled with an uncorrected 90th-percentile threshold (allowing up to 10% false positives per test) and no multiple-comparison control, the field-significance method risks substantial type I error. A matched filtered null, FDR correction, and simulation-based false positive rates are required.

We regret the misunderstanding caused by misleading language in our methodological details. We have now improved our description of our Binary BTSP-like events detection, and state the following:

"*There were two criteria for an event to be considered a possible induction event: during the previous 3 odor presentations the neuron must show no activity within 2 seconds before or after the event in question, and there must not have been a firing field in the top 10% of shuffle data. To determine the significance of a firing field, we took 6-second periods of all previous odor presentations and found the peak of the average activity. We then circularly shuffled each odor presentation and found the peak of the average activity from this shuffled data. This was repeated 2000 times to generate 2000 peak values from shuffled data. For a possible induction event, the real peak of average activity must not have been greater than the 90th percentile of the shuffle. This threshold was chosen to be extremely conservative by excluding all events that are preceded by activity in the top 10% of shuffled data. If an event passed the previous criteria, we considered it as a possible induction event. We then analyzed if it was successful in forming a field. There were four criteria for a successful field formation: 1. The resulting field must have been significant above the 95th percentile of the shuffle; 2. The resulting field occurred within 2 seconds of the peak of the induction event; 3. The neuron must have fired (have value above 2 STD) within 0.5 seconds of the resulting field for the next 3 odor presentations; 4. The neuron must have fired within 0.5 seconds of the resulting field for at least 7 out of the next 10 odor presentations. The strict criteria for activity in the previous 3, following 3, and following 10 odor presentations improves the likelihood that the event in question does induce a stable field. The ± 2 second window for the difference between the event peak and field peak allowed us to look for asymmetrical formation without any bias. The lack of any*

criteria regarding the amplitude of the induction event allowed us to probe the relationship of amplitude to success rate and asymmetrical formation."

One form of our analysis uses thresholds to split the induction events into two groups: 'successes' and 'failures'. We based this analysis on previous published work on spatial BTSP (Grienberger & Magee, 2022) and modified the criteria to fit our olfactory DNMS task with binary categorization of odor-fields as in previous work in our lab using this task (Taxidis et al., 2020). Of note, we made the criteria for detection of BTSP-like events even more conservative than this previous study, since we can record from many more neurons.

We required the 'possible induction event' to have occurred within trials 11-90 because 10 trials before and after are needed for the analysis. No data is filtered out; we simply do not include analysis of induction events that occur in the first or last 10 trials because we need 10 trials before and after for our criteria. This requirement of trials before and after the induction event is consistent with previous spatial BTSP research that exclude events in the beginning and/or end of recording sessions (Grienberger & Magee, 2022; Sumegi et al., 2025). The 90th percentile threshold is used only when finding possible induction events that occur after quiet periods without odor information. We chose the 90th percentile as being more conservative than the 95th percentile because we exclude from analysis all induction events that have an odor field greater than the 90th percentile in the 10 trials **preceding** the induction event. Once an event passes criteria for being a possible induction event with minimal activity in the preceding trials, we continue with a separate step for determining odor field formation.

Our detection for odor field formation is very conservative as well. As compared to previous analysis such as Grienberger and Magee, 2022, who only use 40% reliability in the following 5 trials, we use a **95th** percentile cutoff for the shuffle and also require 70% reliability in the following 10 trials. Once again, the extremely minimal number of 'successes' from small events represent the baseline level or noise floor for our analysis.

Despite our confidence in these conservative criteria, we agree that binarizing events is inherently difficult and are dependent on threshold settings. While this binarized analysis is useful for comparing our work with published spatial BTSP work, we recognize that non-binarized analysis is also necessary. Therefore, we developed a threshold-free analysis in which we compare firing rate increases across sets of trials before and after an induction event (Figure 1 L-N). We find a consistent result across all possible sets of trials used for this analysis (Supplementary Figure S6 A-B). For all experiments of learning and EC silencing, we include now both the binary event detection and parameter-free firing rate increase measure.

Selective pre-exclusion of recent activity

Requiring no activity ±2 s in the three preceding odor presentations excludes neurons with ramping or overlapping events, biasing the sample toward "clean" silent-to-active transitions. This pre-selection may inflate apparent specificity and overlook biologically relevant induction contexts. The impact of this filter must be quantified and, if necessary, replaced with a continuous covariate rather than binary exclusion.

The binary event detection serves to demonstrate the rapid formation of odor-fields in a single trial, aligning with the classic definition of BTSP and with previous BTSP research focused on large somatic events preceded by laps with minimal activity. However, we agree with the reviewer that setting thresholds binarizes the analysis. To address this, we developed the aforementioned threshold-free firing rate increase measure where no pre-exclusion of recent activity is imposed.

Holographic induction paradigm limitations

Single-cell optogenetic stimulation results are shown for very few neurons and mice, with no clear reporting of total stimulation attempts or failure rates. The use of Kv2.1-targeted ChRmine biases activation to proximal compartments, likely differing from natural tuft-driven plateaus. Negative controls (sub-threshold, off-epoch, opsin-negative) and direct comparison of evoked versus spontaneous event waveforms are essential before claiming sufficiency for field writing.

We agree that these optogenetic stimulations are likely different from naturally occurring events; however, we attempted to replicate the standard in the spatial BTSP field, where current is injected into the soma during whole-cell recordings to strongly depolarize the cell. The Kv2.1-targeted ChRmine approach strongly depolarizes the soma in a similar way. In addition to the histograms of all events, we have now included detailed numbers of attempts and successes in the results section. We have now also included numbers of stimulation attempts that occurred outside the odor period and when stimulation did not yield an observable calcium response (opsin-negative or non-responsive). We find that only large calcium events > 10STD preceded odor field formation. No fields were formed following weak responses, non-responses, or strong responses beyond one second after the odor.

Additionally, we now include a new supplementary figure (Supplementary Figure S3, also included below) that compares the shapes and sizes of spontaneous and stimulated events. These results align with other works characterizing template shape and size of somatic calcium events in response to dendritic events (O'Hare et al., 2025). Our large somatic deconvolved events are extremely rare, have larger and longer Delta F/F traces. Our optogenetic stimulation generated a range of responses from 0 to 0.6 Delta F/F. The durations as measured by the full-width at half-maximum of average traces were similar to spontaneously occurring events (between 1 and 1.5 seconds). The result section now states:

"*Using these same criteria as above, we isolated stimulation events that were preceded by minimal activity and no significant field (111 out of 857 total stimulations across 16 recording sessions in 3 mice). Stimulation-induced 'plateau-like' events during the odor cue or ensuring 0.5 seconds had an 11% probability of odor-field formation (5 out of 44 events), similar to that of spontaneous plateau-like events (~8%). In contrast, stimulation-induced 'plateau-like' events outside of this time window (0 out of 33 events), or those with <10 STD amplitude (0 out of 34 stimulation attempts) were not followed by field formation. Importantly, the success rate of field formation and firing rate increase after stimulation (Fig. 2e-f) were similar to the spontaneous events we recorded in Figure 1. Therefore, large somatic plateau-like events causally drive the formation of odor-fields similarly to our observations of spontaneously occurring events.*"

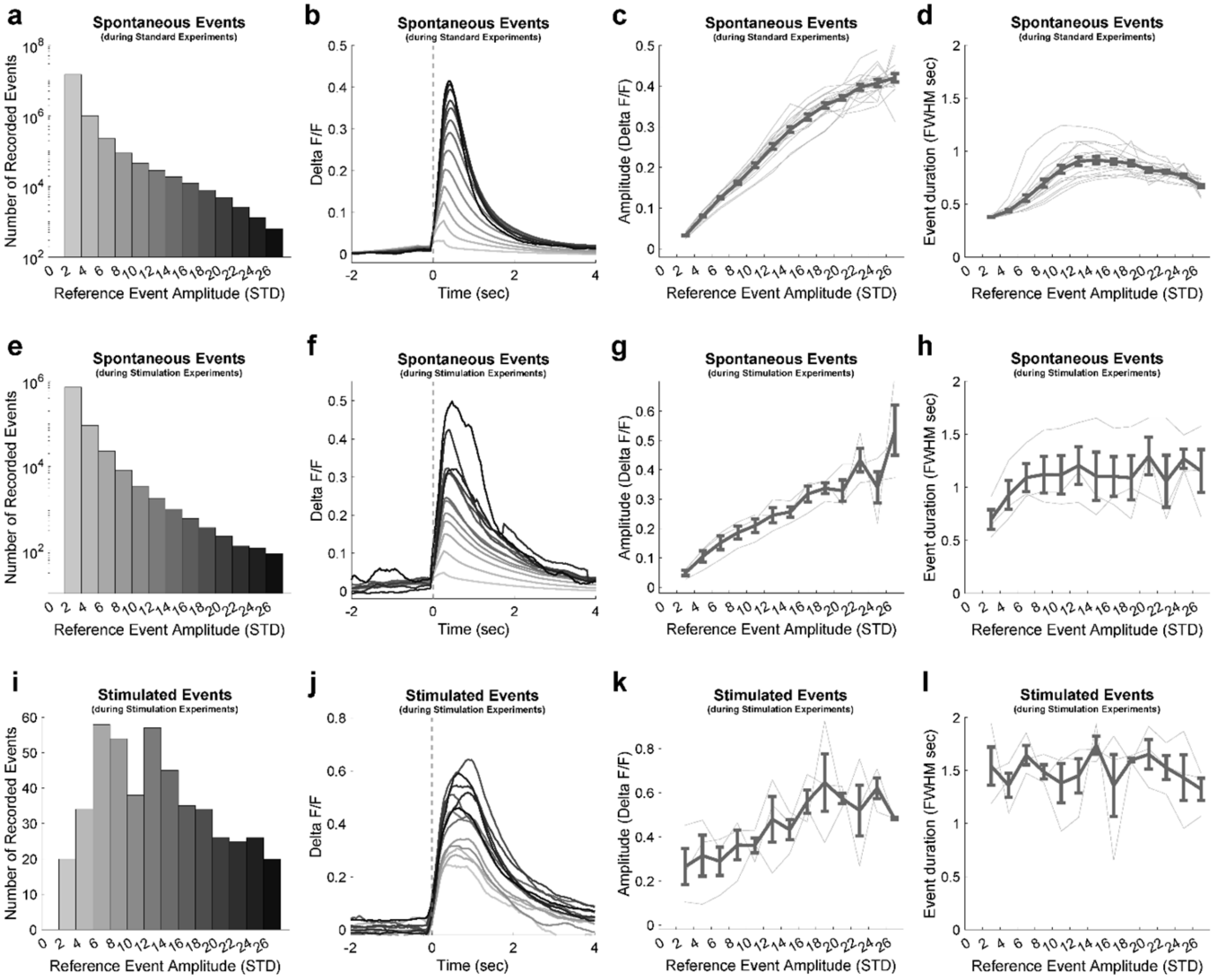

Supplementary Figure S3: Shapes and sizes of 'plateau-like' events. a, sum of total number of recorded deconvolved spontaneous events in the 17 animals from Figure 1 (136 recording sessions). Logarithmic scale shows rare occurrence of large events compared to smaller events. **b,** Delta F/F traces for all events binned into the same deconvolved STD values as in (a) and aligned to the start of the deconvolved event. Colors are matched with (a). **c,** Peak amplitude of average Delta F/F traces from (b). Each line represents one of the 17 animals. Error bar line plot represents mean and SEM. **d,** Full-width at half-maximum (FWHM) for average Delta F/F traces from (b). Lines and error bars are the same as (c). **e-h,** Same as (a-d), but for all spontaneous events recorded during holographic optogenetics experiments from Figure 2 (3 animals, 16 recording sessions). **i-l**, same as (e-h), but only for optogenetically stimulated events from Figure 2. Standard y-axis for (i) instead of logarithmic.

Absence of oscillatory-phase context
BTSP and dendritic excitability are known to be gated by theta phase. Without LFP recordings or phase analysis, the role of rhythmic coordination is speculative. The authors should either provide phase-locking data or explicitly limit mechanistic conclusions.

The reviewer raises an important point that warrants further investigation but is beyond the scope of our current study. To address it here, we adjusted our Discussion and added the following statement about the importance of this rhythmic coordination for future BTSP research:
"*Furthermore, spatial BTSP and dendritic excitability is known to be gated by theta phase, so future experiments must evaluate this relationship across a variety of tasks and types of stimuli.*"

Reviewer #1: Minor Points:

Limited odor set: Only two odors are tested. Generalization to richer odor spaces requires additional stimuli or tempered scope.
Adding more odors to study BTSP in response to more complex sensory stimuli is a very interesting direction but would require a different behavioral setup and training protocols. This goes beyond the scope of the current project which is to describe BTSP-like events outside of a spatial task. However, to address this point, we have adjusted our claims to more closely follow the actual experiments conducted. We also include this new statement in the Discussion:
"*Additionally, our task only uses two different odors, so future work should expand this work to other odor spaces and non-spatial stimuli.*"

Temporal window choice: The use of a fixed 6 s window and occasional 1 s pre-event bins is never justified; analyses should demonstrate robustness to different window lengths.
We used a 6 second window and 95th percentile for odor-field detection to remain consistent with previous research of odor fields in this exact task from our lab (Taxidis et al., 2020). This detail is now included in the text. Again, our threshold-free analysis finds firing rate increase ramps that do not rely on any window.

Anatomical sampling: CA1 exhibits proximodistal and radial innervation gradients from LEC versus MEC; imaging coordinates should be reported and included as covariates.
We agree that this information is important to include. We now state this more clearly in the methods section:
"*A single field of view (FOV) of size 500x500 µm was imaged for all recordings. To maximize SNR, FOVs were selected roughly in the center of our window implants (which were centered around the right dorsal CA1 coordinate 2.0 mm posterior and 1.8 lateral to bregma).*" The same language is repeated for axon imaging but changed to reflect the smaller FOV size of 250x250 µm.

Potentially wrong depiction of errorbars: carefully check errorbar sizes in the figures, most obviously in Fig4c/g; large variance groups seem to have identical or smaller errorbars compared to low variance groups, despite comparable sample size.
We thank the reviewer for noticing this issue. Due to a coding error, we had plotted the same standard error value for both saline and uPSEM groups. We have now updated Figure 4b,c,e,f,g,i, Supplementary Figure S4i,j, and Supplementary Figure S7a with the correct error bars. All other statistics and visualizations were not affected by this mistake.

Terminology clarity: Clear operational definitions for "odor field," "odor cell," "plateau-like event" and "success rate" should be provided in Methods.

We now include these clear definitions in the Methods section “Binary BTSP event detection and analysis”. In the methods, it now reads:
“*We use the following operational definitions:*
‘plateau-like’ event: any recorded somatic calcium event greater than 10 STD.
‘small event’: any recorded somatic calcium event smaller than 10 STD.
‘possible induction event’: an event of any size that satisfies the first 2 criteria: during the previous 3 odor presentations the neuron must show no activity within 2 seconds before or after the event in question, and there must not have been a firing field in the top 10% of shuffle data.
‘odor-field’: a significant activity peak above the 95th percentile of the shuffle
‘success’: a possible induction event of any size that is followed by an odor-field and satisfies 3 other criteria: the resulting field occurred within 2 seconds of the peak of the induction event; the neuron must have fired (have value above 2 STD) within 0.5 seconds of the resulting field for the next 3 odor presentations; the neuron must have fired within 0.5 seconds of the resulting field for at least 7 out of the next 10 odor presentations.
‘failure’: any possible induction event that does not satisfy all the criteria for a ‘success’.
‘success rate’: percentage of all possible induction events that are successful.
‘BTSP-like’ event: any ‘plateau-like’ event that is a ‘success’.”

Causal language: Phrases such as “plateaus drive fields” should be replaced with “precede” or “are associated with” unless necessity is demonstrated.
We have adjusted the language used throughout the manuscript to more closely reflect our findings. For example, in the Results, we now state: “*A single somatic calcium ‘reference event’ was considered an induction event if it preceded the formation of an odor-field.”* Also, “*formation of odor-fields occurred with non-spatial BTSP-like events that are characterized by an asymmetric firing rate increase ramp.”*

Figure legends: Denote whether n refers to neurons, events or mice; show per-mouse data points where possible.
For all figures, we now include these details in the figure legends. All suitable panels were already displaying individual mouse lines and dots, but this has now been made clearer for the reader.

Threshold justification: Percentile cutoffs for induction and field detection (90th/95th) must be rationalized or chosen via ROC/FDR frameworks.
As mentioned in response to a previous point, we based these criteria to match previous publications (Grienberger & Magee, 2022; Taxidis et al., 2020) with our BTSP-like event and odor field detection. The 95th percentile was used exactly as done in previous publications (Taxidis et al., 2020), and the 90th percentile is the more conservative value to discard cells that already had an odor field in the top 10% of a shuffle distribution. The main text and methods text now includes these details for justification.

Reviewer #2: Summary:
The study by Dorian et al. investigates whether behavioral timescale plasticity (BTSP) can drive the formation of memory-related non-spatial hippocampal representations. This question is addressed by performing calcium imaging from hippocampal CA1 cells as well as from LEC and MEC axons in CA1 while mice are performing an olfactory working memory task. In addition, optogenetic induction of BTSPs in CA1 and chemogenetic manipulations of LEC and MEC are performed to establish that

BTSPs are causal in generating odor-related responses and that LEC and MEC are contributing to the success and frequency of BTSPs. Furthermore, recordings during task acquisition show that the relative frequency of BTSP is increased early in training on the odor task. The series of experiments is performed with high rigor, and appropriate statistical methods are used. Importantly, the experiments yield a highly novel result, which is that BTSP is a general plasticity mechanism and not limited to the formation of spatial fields. In addition to reporting this key result, the study also characterizes some mechanism of how BTSPs contribute to the encoding of non-spatial representations. As with any novel phenomenon, the study of course raises a number of interesting questions for future work, such as the limited effectiveness of BTSPs to generate fields later in the delay. The study is nonetheless comprehensive as well as exceptional in its impact and novelty. There are only a few minor points that, if addressed, would further improve the manuscript.
We thank the reviewer for their positive words and careful review of our manuscript. Overall, the reviewer's comments led to additional analysis and improved language that contribute to a stronger manuscript.

Reviewer #2: Minor Points:

(1) The sentence in the abstract 'Using holographic stimulation … ' is somewhat imprecise because spontaneously-occurring events do not need stimulation.
We agree. We have adjusted this sentence in the abstract to say:
"*Using two-photon calcium imaging of CA1 pyramidal neurons in mice learning an odor-cued working memory task, we find spontaneously-occurring plateau-like calcium events during odor cues, forming stable odor representations. These BTSP-like events were more frequent during early stages of task learning, suggesting that BTSP is important for encoding novelty during learning. Additionally, using holographic optogenetics, we induced plateau-like calcium events which were followed by stable odor representations.*"

(2) Fig. 1c. The blue bars are too small to be legible.
We have made the bars wider and brighter.

(3) Panels in Fig. 1g, S1 and S2 seem to show BTSP events later in the delay periods that do not result in fields. Although the low effectiveness of these events to result in fields is pointed out in the text, this could also be more explicitly labelled in the figure panels and mentioned in the figure legends.
We have added an extra arrow in Figure 1g and described the 'failures' in the legends of each figure.

(4) Fig. 1i and S3. Although the results that small events rarely form odor fields is clear, it is less obvious why the timing of the small events shows a value around zero distance from the reference field. If these were completely random events there should at the minimum be an increase variance because the event time and field time are unrelated. Is it possible that 'small events' correspond to an odor fields that occur at a low rate, but nonetheless repeatedly around the same time? Perhaps this point is so obvious that it is not explicitly mentioned. It would nonetheless be helpful for non-experts to provide some explanation for the zero distance other than referring to randomness.
The reviewer raises an important point that we now clarify with the following statements in the Results section:
"*In contrast, only 0.28% "small events" (<10 STD amplitude) during the odor cue or the ensuing 0.5 seconds (254 out of 91,635 total events) preceded new odor fields (Supp. Fig. S5a), These events represent the noise floor of our detection criteria and the rare odor-field formation that formed without*

a preceding 'plateau-like' event. These rare fields were symmetrically distributed around the reference event timepoint with increased variance (Supp. Fig. S5b)."

(5) Fig. 3 d, e, g and i, Fig. S4f and g, Fig. S6b, d, f and h. All the correlations with performance % (or the lack thereof) seem to be a somewhat trivial consequence of effects occurring (or not occurring) early in training. Performance is obviously lower early in training, and any effect that varies with training days would thus necessarily also show a correlation with performance. If the question is asked whether performance is a key predictor in addition to training days, one would have to do much more sophisticated analysis than a simple regression. The relative effect of these two variables (training day and performance) would need to be dissociated, for example by a partial correlation or an analysis of variance that considers the relative contribution of each of the two factors (training day and performance). Appropriate statistical methods should therefore be used, or all of the panels plotting a variable against performance % should be removed along with the corresponding text.

We are thankful to the reviewer for their great suggestion. To disentangle the contribution of novelty versus performance, we have reanalyzed the relevant data using a linear regression model that is equivalent to an ANOVA with continuous variables. We can isolate the effect of day or performance on each measure of BTSP-like events. We now display the t and p values associated with only the effect of performance for all scatter plots (Figure 3d,e,g,h,I, Supplementary Figure S4f,g, and Supplementary Figure S6b,d,f,h). Additionally, we include the results of each linear regression model in Supplementary Tables 1-11 to demonstrate the relative effect of day, performance, and the interaction between the two factors. Although there are changes from the originally displayed p-values, our overall conclusions about the combined effect of day and performance are unaffected.

(6) The use of the term 'odor axon' for MEC axons can be somewhat misleading. While the activity of these axons is clearly timed with regard to stimulus onset and offset, few have selectivity for a particular odor. For comparison, we would not use the term 'place cell' if a cell were not selective for a particular place.

We agree and have adjusted the figures and text to note axons that have their peak activity during the 1st odor, odor offset, or delay period. For example, the results section now states:

"*A much higher percentage of MEC axons had significant peaks during odor presentations and their offset compared to LEC axons (MEC with 28.0 ± 6.5% axons with activity peaking during odor presentations compared to only 9.5 ± 6.5% for LEC; MEC with 4.6 ± 2.2% axons with activity peaking during odor offset compared to only 2.7 ± 1.0% for LEC) (Fig. 5h). MEC axons with activity peaking during odor presentations also showed greater trial reliability compared to LEC (Supp. Fig. S10d). In contrast, a higher percentage of LEC axons had significant activity peaks during the delay period (LEC with 5.8 ± 1.3% axons with activity peaking during delay period compared to only 2.4 ± 0.7% for MEC).*"

(7) The very last phrase in the Results section 'being relayed indirectly through the CA1 perforant path as well' does not sound correct. First, the sentence seems to focus on LEC inputs, but the perforant path through DG and CA3 would be conveying merged MEC and LEC inputs. Second, there is no perforant path with a direct connection to CA1 and the meaning of a 'CA1 perforant path' is therefore not evident.

We fixed the error in the wording to state "… *being relayed indirectly to the CA1 via the perforant path as well*"

(8) In the Results and in the Discussion, it would be more precise to distinguish two potential types of effects as a consequence of manipulating entorhinal inputs to CA1. One type is plasticity/BTSP-related, and although detectable in well-trained mice, may no longer be as behaviorally relevant as it would be if the manipulation were done earlier in training. The second type are effects on hippocampal activity patterns as a consequence of loss of information from LEC or MEC. With any manipulation of a brain region, effects on plasticity and from loss of inputs to connected circuits can often not be fully dissociated. It is nonetheless important to conceptually distinguish between these types of effects and to acknowledge that both contribute to differences in neuronal firing patterns during/after manipulations.

We now discuss the difficulty of teasing these two potential effects apart in the Results:

"*Collectively, LEC inhibition strongly decreased odor-selectivity and decodability in pyramidal CA1 neurons, which may have played a role in the reduction in the success rate of 'plateau-like' events in generating odor-fields, though additional mechanisms could potentially contribute to this effect. Additionally, these results are limited to expert performance, so we are unable to determine if these manipulations would affect the previously found novelty and learning results*."

We discuss it in more detail in the Discussion:

"*First, it is important to note that we only observed these effects in expert-performance. Perhaps the effects that we observed in expert animals would become behaviorally relevant during early training. Alternatively, the loss of information from LEC or MEC may affect CA1 activity regardless of the stage of behavioral performance. Future experiments will be necessary to tease apart these possibilities.*"

Reviewer #2: (Remarks on code availability):

I have reviewed the files and all details for running the code seems to be provided. However, I do not have the resources to run the code and did not install it. The authors refer to large data files in their code. It should perhaps be more explicitly states whether the data files will be uploaded to a repository or are available upon request.

We thank the reviewer for taking the time to evaluate the code. We have improved language in our Data and Code Availability statement:

"*Analysis code for reviewers is attached in the supplementary files. Analysis code will be made available on GitHub after manuscript acceptance. All pre-processed data files used to generate figures will be made publicly available on Dryad after manuscript acceptance. Any additional information necessary to reanalyze the data reported in this paper is available from the lead contacts.*"

REFERENCES

Grienberger, C., & Magee, J. C. (2022). Entorhinal cortex directs learning-related changes in CA1 representations. *Nature*, *611*(7936), 554–562. https://doi.org/10.1038/s41586-022-05378-6

Milstein, A. D., Li, Y., Bittner, K. C., Grienberger, C., Soltesz, I., Magee, J. C., & Romani, S. (2021). Bidirectional synaptic plasticity rapidly modifies hippocampal representations. *eLife*, *10*, e73046. https://doi.org/10.7554/eLife.73046

O'Hare, J. K., Wang, J., Shala, M. D., Polleux, F., & Losonczy, A. (2025). Distal tuft dendrites predict properties of new hippocampal place fields. *Neuron*, *113*(12), 1969-1982.e7. https://doi.org/10.1016/j.neuron.2025.03.029

Priestley, J. B., Bowler, J. C., Rolotti, S. V., Fusi, S., & Losonczy, A. (2022). Signatures of rapid plasticity in hippocampal CA1 representations during novel experiences. *Neuron*, *110*(12), 1978-1992.e6. https://doi.org/10.1016/j.neuron.2022.03.026

Sumegi, M., Olah, G., Lukacs, I. P., Blazsek, M., Makara, J. K., & Nusser, Z. (2025). Diverse calcium dynamics underlie place field formation in hippocampal CA1 pyramidal cells. *eLife*, *13*. https://doi.org/10.7554/eLife.103676.2

Takahashi, H., & Magee, J. C. (2009). Pathway Interactions and Synaptic Plasticity in the Dendritic Tuft Regions of CA1 Pyramidal Neurons. *Neuron*, *62*(1), 102–111. https://doi.org/10.1016/j.neuron.2009.03.007

Taxidis, J., Pnevmatikakis, E. A., Dorian, C. C., Mylavarapu, A. L., Arora, J. S., Samadian, K. D., Hoffberg, E. A., & Golshani, P. (2020). Differential Emergence and Stability of Sensory and Temporal Representations in Context-Specific Hippocampal Sequences. *Neuron*, *108*(5), 984-998.e9. https://doi.org/10.1016/j.neuron.2020.08.028

Response to Reviewers:

We thank Reviewer #1 for their additional constructive feedback and acknowledgement of our "detailed rebuttal and substantial revisions". We have updated the manuscript and addressed all concerns in the point-by-point discussion below.

Reviewer #1:

Thank you for the detailed rebuttal and substantial revisions. Several of my earlier concerns are now at least partially addressed, especially the change in terminology to "plateau-like" events, the added discussion of somatic imaging caveats, and the corrections to error bars. However, I still feel that the strength and scope of the main claims exceed what the data can support, and I would strongly encourage further tempering of the mechanistic and causal language, as well as clearer acknowledgment of alternative interpretations.

We thank the reviewer for their thoughtful feedback. We have now addressed these concerns by improving our statistical tests and further tempering mechanistic and causal language while more clearly discussing alternative interpretations of our results.

Mechanistic and causal claims

While the authors have removed some explicit causal phrases and now refer to "plateau-like" rather than "BTSP-like" events, the title, abstract, and discussion still largely present the results as demonstrating that BTSP is a general plasticity mechanism for non-spatial representations. In particular, the concluding statement in the abstract that the findings "demonstrate a causal role of BTSP" is not supported by the present data. Given that all inferences are based on somatic calcium signals and an operational definition of ≥10 SD events, this remains too strong. The same somatic signature could arise from different underlying mechanisms (e.g. bursts, mixed dendritic/somatic events), and the data demonstrate correlation and temporal precedence rather than necessity or sufficiency. I would recommend consistently rephrasing to something more neutral, such as "rare, large somatic calcium events consistent with BTSP models can precede the formation of some odor fields in this task," and avoiding language that implies that BTSP per se has been established as the mechanism.

We have made several changes to address these concerns.

First, we have changed the Title to read: *"Rapid formation of non-spatial hippocampal representations consistent with behavioral timescale synaptic plasticity is modulated by entorhinal input"*

Second, we have changed the concluding sentence in the Abstract to read: "Together, our findings demonstrate that rare large somatic calcium events, consistent with BTSP, precede and drive novel odor representations during learning, in a manner differentially regulated by medial and lateral entorhinal cortex."

Third, to be consistent with this new language, we have also adjusted similar summarizing sentences in the Discussion.

Last, while we agree that tempering claims is appropriate in regard to the mechanism underlying the formation of odor representations, we would like to point out that experimental results shown in Figure 2 suggest sufficiency since we were able to optogenetically-induce odor representations with somatic excitation. We now acknowledge that BTSP is likely just one of several mechanisms potentially driving formation of hippocampal representations. Furthermore, we now more fully discuss the difficult problem of testing necessity of BTSP in the Discussion.

Operational definition and circularity
The revised text better documents numerators/denominators and threshold choices, but the core concern about circularity remains. “Possible induction events” are defined using stringent criteria (low prior activity, no strong field in the preceding trials, restricted trial window), and “success” is then defined as a new, reliable field emerging near the event in time. This effectively preselects events in which a silent-to-active transition occurs with the desired temporal asymmetry and discards cases where fields emerge more gradually, overlap existing activity, or arise without a qualifying event. The binary analysis therefore builds the BTSP-like pattern into the inclusion criteria and will, by construction, emphasize one-shot, asymmetric field formation. The more continuous “threshold-free” analysis is a welcome addition, but the manuscript should more clearly acknowledge that the binary/“induction” analysis is conditional on these strong filters and cannot be taken as an unbiased estimate of how often plateau-like events genuinely drive fields versus other scenarios.

We have now further revised the text to emphasize the differences between the two types of analysis. In the Results (page 4), it now reads *“Categorizing BTSP-like events into successes and failures restricted analysis to a narrowly defined type of plasticity event based on formation of significant odor responses. Some responses were reinforced by multiple ‘plateau-like’ events (Supp. Fig. S2). Therefore, we developed a simpler analysis to avoid artificially imposed criteria and sensitivity to binary thresholding.”*

In the Discussion, it now reads *“analysis that binarizes complex dynamics into successes and failures is sensitive to chosen thresholds, and this makes it inherently difficult to compare across contexts and recording methods. Similar discrepancies can even be seen within spatial tasks, as the reported percentages of CA1 pyramidal neurons that are classified as place cells can vary dramatically between 20% and 90%*[50]*. Therefore, our reported values for BTSP success cannot be extrapolated to BTSP rates beyond our experimental and analytical parameters. For this reason, we also developed and utilized the threshold-free analysis of firing rate increase.”*

As before, we argue that the binary analysis is still useful because of its widespread use in spatial BTSP research classifying the formation of place fields, but we have now tempered our explanations in the Results and Discussion.

Generality and time representations
The manuscript still suggests that BTSP underlies non-spatial representations more broadly, including time. However, the data show that only a very small minority of plateau-like events occurring after odor offset precede time fields, and optogenetic induction in the delay period did not reliably generate fields. The discussion now notes that delay-related effects are weaker and rare, but the claims about generalization to time and “internally generated” codes still read as if they are an established extension of the mechanism rather than an open question. I would recommend explicitly stating that the present data provide only limited support for BTSP-like contributions to time fields and that the role of such events in temporal coding remains unresolved.

We agree with the reviewer. The noted section in the Discussion now reads *“Although we observed small firing rate increases and rare BTSP-like events that form odor-specific fields during the delay period, our findings only provide limited support of BTSP-like events contributing to temporal coding. Future studies should investigate if recurrent CA3 networks capable of generating temporal codes*[55–57] *can be activated coincidentally with LEC and MEC to drive BTSP for internally generated representations.”*

Behavioral relevance and EC silencing

The chemogenetic manipulations clearly modulate plateau-like event rates, success probabilities, and odor decoding in CA1, but have no detectable effect on behavioral performance in the expert animals. This is now documented more clearly, but the manuscript still leans toward a causal interpretation in which BTSP-dependent representations are central for task solution. Given the lack of behavioral effect in the regime actually recorded, a more balanced formulation would be that LEC and MEC manipulations alter neural representations and event statistics during expert performance, but their necessity for behavior under these conditions is not demonstrated. Any stronger inference about "BTSP creating behaviorally relevant representations" should be softened accordingly or explicitly framed as a hypothesis for future work (e.g. during acquisition or more demanding variants of the task).

We have removed statements that mention 'behavioral relevance' in the Results and Discussion. Additionally, we have expanded the section in the Discussion to further discuss this important idea: *"Perhaps the effects that we observed in expert animals would be stronger if tested during early training. Alternatively, the loss of information from LEC or MEC may affect CA1 activity regardless of the stage of behavioral performance. Furthermore, the lack of behavioral performance deficits with either LEC or MEC inhibition suggests that these representations are not necessary for the performance of this specific task. Future experiments with inhibition at different stages of learning and across various tasks will be necessary to isolate the behavioral relevance of LEC and MEC inputs driving BTSP-like events in CA1."*

Statistical issues

- The authors state that they now use linear regression to separate day and performance, and they have corrected the chemogenetic error bars, which is appreciated. Nonetheless, several statistical concerns remain:
- Regression analyses treat each animal–day as an independent data point without modeling mouse as a random effect. With only five mice followed over multiple days, this violates the hierarchical structure of the data and likely underestimates uncertainty on day/performance effects.

We thank the reviewer for pointing out this error and have now updated all regression figures, statistics, and supplemental tables after modeling mouse as a random effect (slope and intercept). This approach is better suited to repeated measurements for each mouse, better captures animal variability, has more power, and thus strengthens our findings. All corresponding figure panels now display larger t-values and smaller p-values, including 'Plateau-like' Event Rate in Figure 3D which is now significant but weaker than all other effects. We now note this difference in the text. Supplemental Tables contain all MATLAB Table outputs regarding the model and results.

- Event-level analyses still appear to treat individual events as independent observations in some tests, even though many events come from the same cells and animals, again inflating effective n and p-values.

The reviewer's point applies to Figures 1K and 2D depicting histograms for visualization. All statistics and other figure panels are performed on animal averages or session averages. We denote this in the legend text with statements such as *"Individual thin gray lines represent each of the 17 animals (which are the average for each of their 8 recording sessions)"*. The only other use of individual events is when we summarize total counts in the main text (e.g. *"294 'successes' out of 3876 total events"*), which are summary quantifications without statistics involved.

- The multi-factor ANOVAs in the chemogenetic experiments are based on very small group sizes (e.g. n = 3 controls), where parametric assumptions are fragile and effect sizes or confidence intervals would be more informative than marginal p-values.

These points do not overturn the qualitative trends, but they further argue for conservative statements about effect robustness and for avoiding strong mechanistic or causal claims based on borderline statistics.
We appreciate this suggestion. In each figure legend corresponding to figure panels with three-way ANOVAs we have now included the 95% confidence interval for each significant difference. This is included for Figure 4B, 4C, 4E, 4F, 4G, 4I, and Supplementary Figure S6I.

Context from Sumegi et al.
The authors now cite Sumegi et al. as using somatic calcium to study BTSP-like phenomena. Importantly, that work also shows that large somatic Ca^{2+} events with BTSP-like signatures are neither necessary nor sufficient for place field formation and that diverse calcium dynamics can underlie new fields. This does not contradict the present study, but it reinforces the idea that large somatic "plateau-like" events are one of several possible routes to field formation. In my view, this makes it even more important that the current manuscript frame its main conclusion as the identification of one correlated pathway in a specific non-spatial task, rather than as establishing BTSP as the general mechanism for non-spatial representations.

We have edited the first summary paragraph of the Discussion to read *"These events are consistent with models of behavioral timescale synaptic plasticity (BTSP), as they demonstrate asymmetrical formation observed previously during spatial tasks*[1–11]*. This suggests that BTSP may be one plasticity mechanism capable of driving rapid formation of hippocampal representations during both spatial and non-spatial cognition."*

Additionally, we have expanded a section to discuss the diversity of calcium events as seen in Sumegi et al. It now reads *"Additionally, alternative mechanisms may also play a role, consistent with recent research demonstrating a variety of somatic calcium dynamics preceding formation of place fields*[51]*. Indeed, the rapid formation of odor responses after optogenetically-induced somatic plateau-like events suggests sufficiency, but the low success rate suggests other mechanisms could also be involved. Furthermore, tests of necessity are difficult due to our present limited knowledge of the mechanisms underlying BTSP."*

In summary, the additional analyses and revisions improve the manuscript, but I still recommend a further toning down of mechanistic and causal language, more explicit discussion of alternative mechanisms and the limitations of the operational definition, clearer acknowledgment of the lack of behavioral impact of EC silencing under the conditions tested, and a more cautious treatment of generalization to time fields.

We thank the reviewer for their suggestions. We have addressed each of these concerns with revisions of text throughout the Abstract, Results, and Discussion sections.
